# Omega-3 fatty acids and bioactives in aqueous-extracted oils from seven edible insects

**DOI:** 10.1371/journal.pone.0356807

**Published:** 2026-08-26

**Authors:** Xavier Cheseto, Chrysantus Mbi Tanga

**Affiliations:** International Centre of Insect Physiology and Ecology (icipe), Nairobi, Kenya; Benha University, EGYPT

## Abstract

Globally, there is urgent search and research interest towards alternative sources of polyunsaturated omega-3 fatty acids (ω-3 FA) due to sustainability challenges faced by dependence on fish and plant-derived sources. Limited information exists on insects despite their promising potential. This work analyzed oils from seven insect species commonly consumed globally compared to commercial sesame oil using coupled gas chromatography-mass spectrometry (GC-MS) and liquid chromatography-mass spectrometry (LC-MS). Oil yields from insects ranged between 11.1 mg/g to 115.3 mg/g. Over 67 fatty acids, α-tocopherol [vitamin E] and eight flavonoids were identified. All insect oils contained ω-3 FA [i.e., α-linolenic acid (ALA), eicosapentaenoic acid (EPA), and docosahexaenoic acid (DHA)]. The fifth instar of desert locust [*Schistocerca gregaria*] had the highest levels of ALA (9.4 µg/mg), EPA (1.0 µg/mg), and DHA (3.1 µg/mg) compared to sesame oil with ALA (7.9 µg/mg) only. Polyunsaturated fatty acids were dominant in sesame oil. Over 50% of monounsaturated fatty acids were present in *S. gregaria*, *Spodoptera frugiperda* and *Bactrocera dorsalis*. *Gryllus bimaculatus* oil contained mostly saturated fatty acids (51.1%). The levels of α-Tocopherol [vitamin E] were significantly higher in all the insect oils except *S. frugiperda*. Flavonoids such as quercetin and luteolin were abundantly present in *S. gregaria* and *G. bimaculatus* oils. These insects are widely consumed across the globe and can be farmed using fewer resources. Thus, widening the research scope would offer new opportunities to advance use of edible insects to address interconnected nutritional and health challenges.

## Introduction

Omega-3 fatty acids (ω-3 FA), such as α-linolenic acid (ALA), eicosapentaenoic acid (EPA) and docosahexaenoic acid (DHA), are essential dietary lipids known to support heart health, brain function, mood balance, and may offer anti-diabetic and anti-cancer benefits [1–3]. Their primary sources have traditionally included fatty fish, flaxseeds, chia seeds, and walnuts [4–6]. However, concerns about overfishing, environmental degradation, and resource-intensive crop production are raising concerns about the long-term sustainability of these foods. This has intensified the search for alternative, reliable sources of lipids and ω-3 FA [7,8].

Beyond ω-3 FAs, dietary lipids can serve as carriers of important bioactive compounds, including fat-soluble vitamins and phytochemicals, which contribute to their overall nutritional and functional value [9,10]. Vitamin E (tocopherols), for instance, acts as a lipid-soluble antioxidant that protects cellular components from oxidative damage and supports immune function [11]. In edible insect oils, α-tocopherol concentrations have been reported to range from approximately 2–11 µg/mg of oil, with species such as *Schistocerca gregaria, Ruspolia diﬀerens,* and *Gryllus bimaculatus* showing particularly high levels [12–15]. Similarly, flavonoids, including quercetin, luteolin, and kaempferol, exhibit antioxidant, anti-inflammatory, and cardioprotective effects, with reported levels in insect oils ranging from 0.2 to 1.3 µg/mg of oil depending on the species [14,15]. Despite their recognized importance, these bioactive constituents remain underexplored in many alternative lipid sources.

In this context, edible insects have emerged as promising and sustainable alternatives. They are characterized by low environmental footprint, efficient feed conversion, and ability to thrive on waste streams [16–18]. Beyond their well-known protein content, many insects’ species contain oils naturally enriched with ω-3 FA and other bioactive compounds [8,19], positioning them as an underexplored yet potentially valuable contributor to future dietary lipid sources

While insect protein has been extensively studied and recognized for its high nutritional value, insect oils, the second most abundant nutrient in many species, have largely been overlooked [19,20]. These oils represent valuable byproducts of protein processing and offer considerable potential for application in food, feed, biofuel production, pharmaceuticals, and cosmetics [14,21,22]. Despite this promise, the nutraceutical properties of insect oils, particularly their content of ω-3 FA content, vitamin E and flavonoids remain poorly characterized.

In this study, we assessed the fatty acid and bioactive compound profiles of oils extracted from seven edible insect species representing three orders (Orthoptera, Diptera, and Lepidoptera) commonly consumed across Africa. Oils were obtained using an aqueous extraction method selected for its affordability, environmental benefits, and safety. We quantified and compared oil yields and analyzed (i) fatty acid compositions including essential ω-3 FA, (ii) vitamin E content, and (iii) flavonoid profiles. For *B. dorsalis* adult, *B. dorsalis* L3 and *C. capitata* adult, vitamin E and flavonoid analyses were not performed due to limited sample availability. All results were compared with commercial sesame oil (*Sesamum indicum*), a locally available in Kenya, where its widely cultivated in (western, coastal, semi-arid regions),and primarily used as seed oil valued for its nutritional, economic importance and with a well-characterized profile of fatty acids, vitamin E, and bioactive compounds, making it a suitable reference for evaluating the functional potential of edible insect-derived oils [23,24]. It is also more affordable than most imported oils like sunflower, corn, and palm oils, which are not only costly but also frequently subject to adulteration, much like high-end extra virgin olive oil [25].

## Materials and methods

### Insects

In this study, insects representing three orders (Orthoptera, Diptera, and Lepidoptera), were selected to expand our existing database of characterized oils. Previous analyses from our group include oils from the adult desert locust (*Schistocerca gregaria*), the adult African bush-cricket (*Ruspolia differens*) and termite alates (*Macrotermes* spp) [14,15]. The newly selected species were reared on standardized diets using established protocols and production systems. Insects were harvested at defined developmental stages, as outlined in Table 1. All rearing and sample preparation were conducted at the International Centre of Insect Physiology and Ecology (*icipe*) in Nairobi, Kenya.

**Table 1:** Edible insect species used for oil extraction, including their rearing diets and harvested developmental stages.

Order	Species	Stage used	Rearing diet
Orthoptera	*Schistocerca gregaria* (desert locust)	Fifth instar (L5)	wheat seedlings, maize leaves, wheat bran
	*Gryllus bimaculatus* (African field cricket)	Adult	wheat bran, soybean, fish offal, pumpkin leaf, carrot, maize meal
	*Scapsipedus icipe* (native cricket)	Adult	wheat bran, soybean, fish offal, pumpkin leaf, carrot, maize meal
Diptera	*Bactrocera dorsalis* (oriental fruit fly)	Third instar (L3); Adult	4:1 mixture of sugar (Mumias Sugar Co., Nairobi, Kenya) and enzymatic yeast hydrolysate (USB Corporation, Cleveland, OH)
	*Ceratitis capitata* (Mediterranean fruit fly)	Adult	4:1 mixture of sugar and enzymatic yeast hydrolysate
Lepidoptera	*Spodoptera frugiperda* (fall armyworm)	Fifth instar (L5)	maize leaves

### Plant oil

A 500 mL bottle of cold-pressed, unrefined sesame oil (Chepper industries, Nakuru, Kenya) was purchased from a local supermarket in Nairobi, Kenya.

### 2.3. Extraction of insect oils

For each insect species and the corresponding developmental stage targeted (Table 1), oil was extracted 0.5 kg of wet insect biomass using an aqueous extraction method adapted from previously described methods [14,26]. The insects were killed by freezing at −80 °C for 3 h followed by homogenization in 1000 mL of distilled deionized water for 5 min. The slurry was transferred to a 1.5 l conical flask and heated at 80 °C for 3 h with gentle magnetic stirring, followed by filtration through a double-layered gauze cloth.

The filtrate was transferred into a separating funnel and left undisturbed overnight to allow phase separation. The upper layer was collected, mixed with 0.9% NaCl solution (20 mL) (Sigma-Aldrich, St. Louis, MO, USA) and centrifuged at 14000 rpm for 10 min. The resulting supernatant (insect oil), recovered, weighed and stored at −20 °C until analysis. Three independent extractions were performed for each species using separate insect batches. Oil yield was calculated using the following equation:


$$\begin{array}{c} \text{Insect Oil Yield }(\%)=\left\{\text{Weight of Extracted Oil}|\text{Initial Weight of Insect Biomass }\right\}*\text{100} \end{array}$$

### GC-MS analysis of fatty acids

Fatty acid profiles of the insect oils and sesame oil were determined after derivatization to fatty acid methyl esters (FAMEs) using a sodium methoxide (15 mg/mL in dry methanol; Sigma-Aldrich, St. Louis, MO, USA). Briefly, 100 mg of each sample was mixed with 500 µL of the sodium methoxide solution, vortexed for 10 s, sonicated for 10 min, and then incubated at 60 °C for 1 h. The reaction was terminated by adding 100 μL of deionized water, followed by 10 s vortexing. FAMEs were extracted with 1 mL of GC-grade hexane, centrifuged at 14000 rpm for 5 min, and the organic layer was dried over anhydrous Na_2_SO_4(s)_ (Sigma-Aldrich, St. Louis, MO, USA). An aliquot (1 µL) of each extract was analyzed on a GC-MS system (7890A GC coupled to a 5975 C mass selective detector, Agilent Technologies, Inc., Santa Clara, CA, USA) fitted with a HP5 MS low bleed capillary column (30 m × 0.25 mm i.d., 0.25 µm film thickness; J&W, Folsom, CA, USA. Helium was used as the carrier gas at a flow rate of 1.25 mL/min. The oven temperature was ramped from 35 °C to 285 °C, while the MS ion source and quadrupole were maintained at 230 °C and 180 °C, respectively. A filament delay of 3.3 min was applied. For external quantification, a calibration curve was generated using an authentic methyl octadecenoate standard (≥ 99%, Sigma-Aldrich, St. Louis, MO, USA) across a concentration range of 0.2–125 ng/μL under identical conditions. Compound identities were confirmed based on retention times, authentic standards (where available), and comparison of spectral data with reference spectra from Adams, Chemecol, NIST 05, 08, and 11 libraries. All insect oil samples were analyzed in triplicate, with each replicate derived from an independent extraction batch. FAME concentrations were expressed as µg/mg of oil.

### Extraction and GC-MS analysis of Vitamin E (α-tocopherol)

Analysis of samples for vitamin E (α-tocopherol), was performed following a previously established protocol [14] with minor modifications. Briefly, 300 mg of insect or plant oil was placed into a 10 mL glass vial, after which 5 mL of a solvent mixture (hexane: methanol: deionized water; 2:1:2, v/v) was added. The mixture was vortexed for 10 s, sonicated for 30 min, and centrifuged at 14,000 rpm for 5 min. The resulting supernatant was dried over anhydrous Na_2_SO_4(s),_ evaporated to dryness under gentle stream of N2_(g)_ and any residual fatty acids were derivatized using above mentioned procedure (GC-MS analysis of fatty acids). All analyses were performed in triplicate, with each replicate prepared from an independent insect oil batch.

### Extraction and LC-MS analysis of Flavonoids

Flavonoids were extracted following a previously established protocol [14], with minor modifications. Briefly, 300 mg of each insect or plant oil sample was transferred into a 2 mL Eppendorf tube containing 1,000 µL of LC-MS-grade acetonitrile (Sigma-Aldrich, St. Louis, MO, USA). The mixture was vortexed for 10s, sonicated for 30 min, and centrifuged at 14,000 rpm for 15 min. The resulting acetonitrile-soluble supernatant was filtered through Whatman No. 42 filter paper and collected into a 2 mL autosampler vial for LC-MS analysis.

Chromatographic separation was performed on an Agilent 1260 Infinity HPLC system (Agilent Technologies, Palo Alto, CA, USA) coupled to an Agilent 6120 Single Quadrupole MS detector. Separation done on a ZORBAX SB-C_18_ column (4.6 × 250 mm, 3.5 µm) maintained at 40 °C. The mobile phases consisted of water (A) and acetonitrile (B), each containing 0.01% formic acid. The gradient program was as follows: 0.01 min, 5% B; 0.01–5 min, 5% B; 5–10 min, 5–20% B; 10–15 min, 20% B; 15–20 min, 20–80% B; 20–25 min, 80% B; 25–30 min, 80–100% B; 30–35 min, 100–37 min, 100–5% B; and 37–42 min, 5% B. The flow rate was 0.5 mL/min, and the injection volume was 3 µL.

The single-quadrupole MS was operated in two separate acquisition methods, one in ESI positive mode and the other in ESI negative mode, each scanning across an m/z range of 100–2,000. The instrument was set with the following parameters: capillary voltage, 3.0 kV; cone voltage, 30 V; extractor voltage, 5 V; RF voltage, 0.5 V; source temperature, 110 °C; desolvation temperature, 380 °C; and desolvation nitrogen flow, 400 L/h. Mass spectra were recorded for each chromatographic peak, and compounds were tentatively identified by comparing the spectra with reference databases, including METLIN, ChemSpider, ChemCalc, and the NIST MS/MS libraries.

Quercetin, luteolin, kaempferol, rutin, and apigenin were confirmed through co-injection with high-purity authentic standards (≥ 90–98% purity; Sigma-Aldrich, St. Louis, MO, USA). For quantification, a linear calibration curve (peak area vs. concentration, 1–100 ng/µL) was established for each standard. All measurements were performed in triplicate, with each replicate prepared from a separate batch of extracted oil.

### Data analysis

Data was first entered into Microsoft Excel, and all statistical analyses were performed using R software version 3.5.0 [27]. To illustrate the relative distribution of fatty acids across the insect and plant oils, 100% stacked bar charts were created in Excel. Differences in the proportions of saturated, monounsaturated, and polyunsaturated fatty acids were assessed using Chi-square tests. Concentrations of individual fatty acids, flavonoids, oil yields, and vitamin E were compared using one-way ANOVA followed by the Student–Newman–Keuls (SNK) post hoc test. When the assumption of equal variance was violated, Welch’s ANOVA was applied. Normality and homogeneity of variance were checked using the Shapiro–Wilk test (P > 0.05) and Levene’s test (P > 0.05), respectively. Statistical significance was defined at P < 0.05. Flavonoid concentrations were reported in ng/g of oils as Mean ± standard error (SE) based on triplicate determinations. Data used to generate all the graphs are available from Figshare [28].

## Results

### Insect oil yields

**Fig 1:**
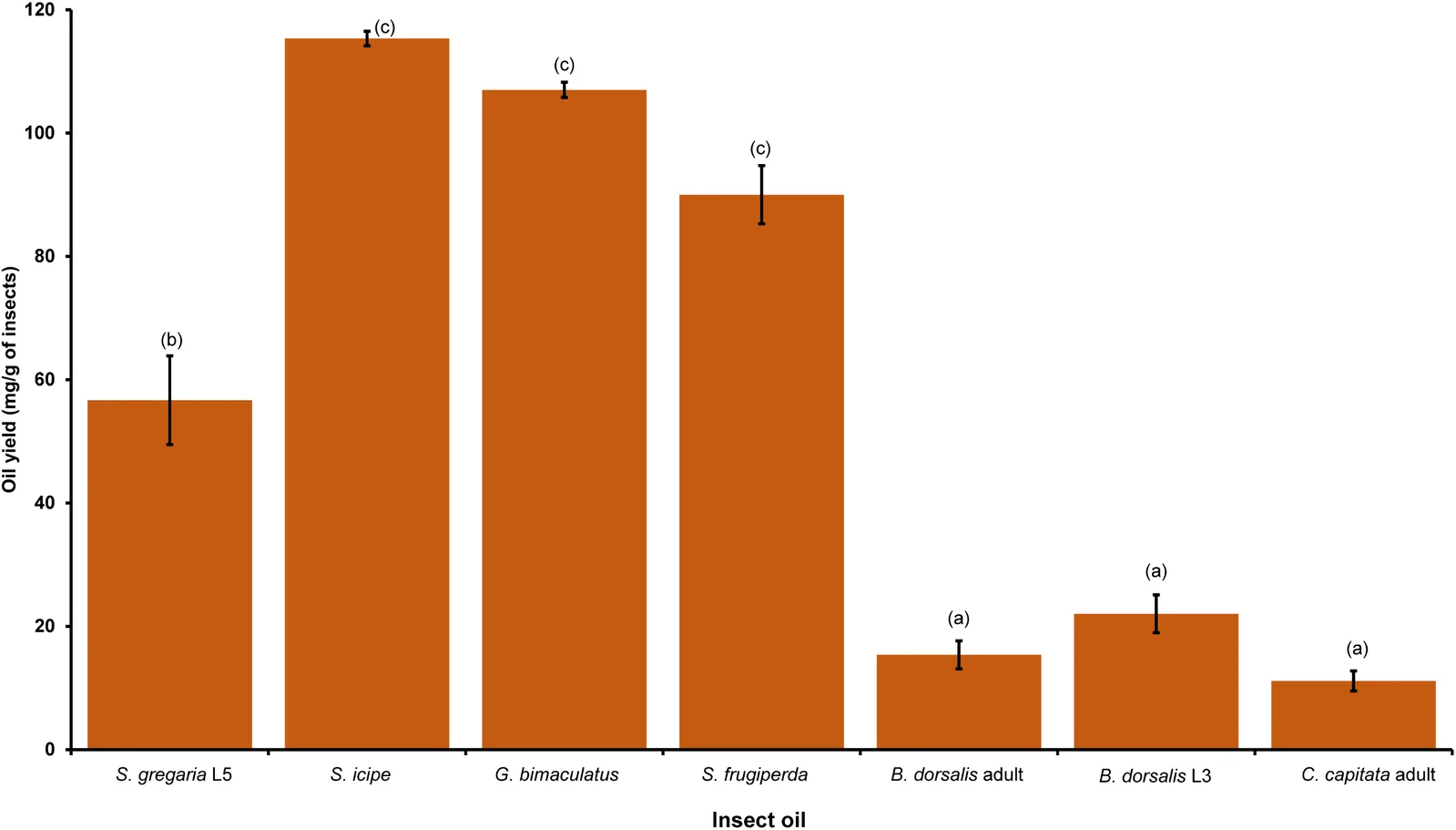
Oil yields seven insect samples. Error bars indicate the standard error. Bar graph with different letters is significantly different from each other (ANOVA followed by Student-Neuman-Keul’s (SNK’s) post-hoc test; *p* < 0.05, *n* = 3).

Oil yields varied significantly across the seven insect species studied (Fig 1). Yields ranged from 11.1 ± 1.63 mg/g in *C. capitata* to 115.3 ± 1.19 mg/g in *S. icipe*. The two cricket species (*S. icipe* and *G. bimaculatus*) produced approximately twice the oil of *S. gregaria* L5 among the orthopterans. The fall armyworm (*S. frugiperda*) yielded 90.0 ± 4.71 mg/g, comparable to the crickets and 1.5 times higher than *S. gregaria* L5. Fruit flies (*B. dorsalis* and *C. capitata*) showed the lowest yields.

### Fatty acid composition of insect and plant oils

Table 2 presents the identified FAMEs in insects and plant oils. A total of 66 FAMEs were identified across the insect and plant oils analyzed, with sesame oil containing 44 FAMEs. Among the insect oils, *S. gregaria* L5 and *G. bimaculatus* registered the highest total number of FAMEs (53 each), followed by *S. icipe* (51). In contrast, oils from *B. dorsalis* (adult, 41; L3, 40) and *C. capitata* (42) had FAME profiles comparable to sesame oil, while *S. frugiperda* contained 34 detectable FAMEs.

**Table 2:** Fatty acid composition (μg/mg of insect oil*) analyzed by GC-MS.

t_R_ (min)	Compound Name	Sesame oil	*S. gregaria* L5	*S. icipe*	*G. bimaculatus*	*S. frugiperda* L5	*B. dorsalis* adult	*B. dorsalis* L3	*C. capitata* adult	F _(7,16)_	*p*-value
13.25^i^	Methyl octanoate	0.5 ± 0.23^a^	0.7 ± 0.09^a^	1.5 ± 0.34^a, b^	0.3 ± 0.05^a^		0.7 ± 0.43^a^	3.9 ± 0.31^c^	2.5 ± 0.39^b, c^	15.66	< 0.001
16.23^i^	Methyl decanoate		0.3 ± 0.17^a, b^	0.3 ± 0.11^a, b^	0.1 ± 0.01^a, b^	0.1 ± 0.01^a, b^	0.3 ± 0.12^a, b^	0.9 ± 0.32^b^	0.6 ± 0.10^a, b^	3.22	0.025
16.93^ii^	Methyl 4,8-dimethylnonanoate	0.6 ± 0.25^a^	0.3 ± 0.06^a, b^							4.01	0.010
17.54^i^	Methyl undecanoate	0.2 ± 0.02^a^							0.3 ± 0.05^a^	19.65	< 0.001
18.85^i^	Methyl dodecanoate	5.7 ± 0.50^a^	0.5 ± 0.04^b^	0.3 ± 0.10^b^	0.5 ± 0.16^b^	0.6 ± 0.19^b^	1.9 ± 0.33^b, c^	2.7 ± 0.53^c^	1.5 ± 0.13^b, c^	23.91	< 0.001
19.25^ii^	Methyl 2,6-dimethylundecanoate		0.2 ± 0.07^a, b^	0.5 ± 0.20^b^	0.1 ± 0.01^a, b^					3.82	0.013
19.60^ii^	Methyl 11-methyldodecanoate	7.9 ± 0.56^a^	0.2 ± 0.06^b^	0.3 ± 0.11^b^	1.5 ± 0.68^b^	0.1 ± 0.01^b^				49.14	< 0.001
19.69^ii^	Methyl 10-methyldodecanoate	0.5 ± 0.11^a, b^	1.5 ± 0.13^c^	0.5 ± 0.18^a, b^	0.1 ± 0.03^a, b^		0.6 ± 0.19^a, b^	0.8 ± 0.19^b, c^	0.4 ± 0.05^a, b^	8.24	< 0.001
20.02^i^	Methyl tridecanoate		0.3 ± 0.07^a, b^	0.2 ± 0.08^a, b^	0.2 ± 0.04^a, b^	0.7 ± 0.13^b, c^	0.5 ± 0.2^a, b^	1.3 ± 0.11^c^	0.2 ± 0.11^a, b^	9.27	< 0.001
20.46^ii^	Methyl 3-methyltridecanoate		0.3 ± 0.10^a, b^	0.1 ± 0.03^a, b^	0.1 ± 0.07^a, b^		0.7 ± 0.21^a, b^	0.6 ± 0.12^a, b^	1.6 ± 0.64^b^	3.22	0.022
20.75^ii^	Methyl 12-methyltridecanoate		2.3 ± 0.89^b^	0.1 ± 0.01^a^	0.2 ± 0.13^a^	0.6 ± 0.16^a, b^	0.2 ± 0.07^a^	0.3 ± 0.13^a^	0.1 ± 0.06^a^	3.50	0.018
21.15^i^	Methyl tetradecanoate	5.5 ± 0.22^a, b^	4.8 ± 0.17^b^	6.7 ± 0.25^a^	4.5 ± 0.63^b^	2.7 ± 0.18^c^				71.30	< 0.001
21.51^ii^	Methyl 2-methyltetradecanoate		0.3 ± 0.05^a^	0.4 ± 0.10^a^	0.5 ± 0.22^a^					3.47	0.190
21.84^ii^	Methyl 13-methyltetradecanoate		0.5 ± 0.04^a^	0.4 ± 0.07^a^	0.3 ± 0.07^a^	0.5 ± 0.22^a^				3.98	0.011
21.93^ii^	Methyl 9-methyltetradecanoate		0.6 ± 0.06^b^	0.7 ± 0.05^b^	0.5 ± 0.12b					26.39	< 0.001
22.23^i^	Methyl pentadecanoate		1.0 ± 0.08^a, b^	1.1 ± 0.04^a, b^	0.4 ± 0.06^a^	0.3 ± 0.08^a^	1.9 ± 0.2^b, c^	5.5 ± 0.42^d^	2.8 ± 0.11^c^	71.37	< 0.001
22.66^ii^	Methyl 3-methylpentadecanoate		0.1 ± 0.07^a^	0.2 ± 0.09^a^	0.1 ± 0.01^a^		0.2 ± 0.07^a^	0.6 ± 0.09^b^	0.1 ± 0.04^a^	7.92	< 0.001
23.25^i^	Methyl hexadecanoate	83.7 ± 0.90^a^	23.8 ± 1.41^b^	34.3 ± 1.55^c^	86.7 ± 2.13^a^	10.7 ± 0.99^d^	2.1 ± 0.87^e^	10.4 ± 1.01^d^	11.4 ± 0.68^d^	468.50	< 0.001
23.60^ii^	Methyl 10-methylhexadecanoate		34.5 ± 1.64^b^	50.4 ± 1.28^c^	22.0 ± 1.65^d^	12.6 ± 1.37^e^	3.5 ± 0.29^a, f^	6.0 ± 0.26^a, e^	6.8 ± 0.94^e, f^	166.00	< 0.001
24.01^ii^	Methyl 14-methylhexadecanoate		0.5 ± 0.13^a, b^	0.3 ± 0.04^a, b^	0.1 ± 0.04a	0.3 ± 0.19^a, b^	0.6 ± 0.22^a, b^	2.1 ± 0.17^c^	1.1 ± 0.12^b^	16.80	< 0.001
24.21^i^	Methyl heptadecanoate	1.2 ± 0.08^a^				0.1 ± 0.01^b^				132.00	< 0.001
24.22^ii^	Methyl 15-methylhexadecanoate		1.5 ± 0.14^b^			0.1 ± 0.02^a^				78.03	< 0.001
25.43^i^	Methyl octadecanoate	52.4 ± 0.26^a^	72.6 ± 0.81^b^	90.3 ± 1.22^c^	28.5 ± 3.34^d^	10.9 ± 1.6^e, f^	5.3 ± 0.45^e^	18.3 ± 0.92^f^	10.6 ± 1.10^e, f^	297.6	< 0.001
25.62^ii^	Methyl 6-methyloctadecanoate	2.7 ± 0.62^a^	0.4 ± 0.02^a, b^	0.3 ± 0.06^a, b^	1.8 ± 0.93^a, b^					4.35	< 0.001
26.09^i^	Methyl nonadecanoate		0.5 ± 0.06^a, b^	0.4 ± 0.08^a, b^	0.8 ± 0.33^a, c^	0.2 ± 0.03^a, b^	0.5 ± 0.19^a, b^	1.1 ± 0.25^b, c^	1.6 ± 0.18^c^	5.83	0.002
26.91^i^	Methyl eicosanoate	3.2 ± 0.42^a^			1.8 ± 0.19^a, c^	0.4 ± 0.07^b, c^	1.8 ± 0.10^a, c^	2.8 ± 0.52^a^	2.1 ± 0.09^a^	15.72	< 0.001
26.91^ii^	Methyl 18-methylnonadecanoate	2.3 ± 0.44^a^								18.7	< 0.001
27.17^ii^	Methyl 2,6-dimethylnonadecanoate		1.0 ± 0.40^b^	0.1 ± 0.02^a, b^	0.2 ± 0.05^a, b^		0.5 ± 0.19^a, b^	0.4 ± 0.05^a, b^	0.1 ± 0.02^a, b^	3.08	0.030
27.73^i^	Methyl heneicosanoate	12.2 ± 0.48^a^	0.1 ± 0.07^b^	0.1 ± 0.04^b^	0.5 ± 0.20^b^		0.7 ± 0.36^b^	0.5 ± 0.09^b^	0.1 ± 0.03^b^	229.5	< 0.001
27.86^ii^	Methyl 2,4,8-trimethylheneicosanoate	0.6 ± 0.28^a^	1.8 ± 0.74^a^	0.1 ± 0.03^a^	0.9 ± 0.48^a^		0.1 ± 0.01^a^	0.1 ± 0.01^a^	0.05 ± 0.01a	2.52	0.060
28.01^ii^	Methyl 3-methylheneicosanoate		0.1 ± 0.07^a, b^	0.1 ± 0.05^a, b^	1.1 ± 0.50^b^		0.1 ± 0.03^a, b^	0.1 ± 0.03^a, b^	0.2 ± 0.03^a, b^	2.72	0.460
28.53^i^	Methyl docosanoate	2.4 ± 0.26^a^	0.4 ± 0.08^b^	0.5 ± 0.04^b^	0.3 ± 0.13^b^	0.1 ± 0.02^b^				36.57	< 0.001
29.29^ii^	Methyl 20-methyldocosanoate	1.9 ± 0.31^a^			0.4 ± 0.08^b^					23.58	< 0.001
30.06^ii^	Methyl 16-methyldocosanoate	2.3 ± 0.29^a, b^					3.4 ± 0.38^a^	7.0 ± 1.08^c^	3.7 ± 0.49^a^	21.71	< 0.001
	Ʃ SFA	185.8 ± 6.50	151.1 ± 7.58	190.1 ± 6.16	154.5 ± 12.33	40.8 ± 5.32	25.6 ± 4.90	65.4 ± 6.62	47.9 ± 5.34		
19.91^ii^	Methyl 12-hydroxy-9-octadecenoate	1.8 ± 0.26^a^								31.35	< 0.001
14.27^ii^	Methyl 2-hexenoate	0.5 ± 0.28^a^	0.2 ± 0.04^a^	0.1 ± 0.02^a^	0.04 ± 0.01^a^		0.04 ± 0.01^a^	0.1 ± 0.02^a^	0.05 ± 0.01^a^	2.040	0.112
20.83^ii^	Methyl (5Z)-dodecenoate	5.5 ± 0.14^a^								264.30	< 0.001
20.88^ii^	Methyl (11E)-tetradecenoate	3.3 ± 0.18^a^	0.3 ± 0.03^b^	0.2 ± 0.05^b^	0.2 ± 0.11^b^		0.4 ± 0.08^b^	0.3 ± 0.04^b^	0.5 ± 0.11^b^	51.50	< 0.001
20.94^ii^	Methyl (11Z)-tetradecenoate	6.0 ± 0.35^a^	0.3 ± 0.06^b, c^	0.3 ± 0.04^b, c^	0.4 ± 0.07^b, c^		0.7 ± 0.09^b, c^	0.6 ± 0.01^b, c^	0.7 ± 0.12^c^	161.90	< 0.001
20.98^ii^	Methyl (9Z)-tetradecenoate	2.3 ± 0.17^a^				0.1 ± 0.02^b^				39.90	< 0.001
23.04^i^	Methyl (9Z)-hexadecenoate	2.8 ± 0.21^a^	1.5 ± 0.07^a^	2.7 ± 0.21^a^	1.4 ± 0.29^a^	1.0 ± 0.11^a^	4.9 ± 0.73^b^	5.2 ± 0.50^b^	5.3 ± 0.22^b^	17.58	< 0.001
23.97^ii^	Methyl 9-heptadecenoate	2.4 ± 0.51^a^								41.66	< 0.001
24.01^ii^	Methyl 8-heptadecenoate	1.1 ± 0.35^a, b^	2.3 ± 0.08^a, b^	3.8 ± 0.15^a, c^	2.2 ± 0.46^a, b^		6.2 ± 0.37^c, d^	7.8 ± 0.79^d^	7.7 ± 0.94^d^	23.52	< 0.001
24.06^ii^	Methyl (10Z)-heptadecenoate		0.9 ± 0.05^a, b^	1.4 ± 0.15^b, c^	1.3 ± 0.21^a, c^	0.4 ± 0.07^a, b^	3.1 ± 0.47^d^	2.8 ± 0.13^d^	2.4 ± 0.2^c^, d	15.82	< 0.001
24.91^ii^	Methyl (6Z)-octadecenoate		0.4 ± 0.06^a, b^	0.4 ± 0.09^a, b^	0.3 ± 0.10^a^		2.0 ± 0.49^c^	1.4 ± 0.05^b, c^	1.0 ± 0.10^a, c^	10.75	< 0.001
24.94^i^	Methyl (9Z)-octadecenoate	182.1 ± 15.68^a^	57.7 ± 1.47^b^	94.8 ± 2.04^c^	80.4 ± 0.79^c^	24.9 ± 2.28^d^	11.0 ± 0.71^d^	16.1 ± 0.36^d^	13.8 ± 0.66^d^	1641	< 0.001
25.24^ii^	Methyl (13E)-octadecenoate	1.1 ± 0.06^a^				0.9 ± 0.15^a^				7.63	< 0.001
25.25^ii^	Methyl (6E)-octadecenoate	0.9 ± 0.27^a, b^	2.6 ± 0.09^a, c^	3.9 ± 0.28^c^	2.1 ± 0.77^b, c^		7.4 ± 0.46^d^	9.8 ± 0.31^e^	8.7 ± 0.32^d, e^	61.96	< 0.001
25.31^ii^	Methyl (16Z)-octadecenoate	1.1 ± 0.08^a, b^	24.5 ± 0.63^c^	40.4 ± 1.20^d^	30.5 ± 2.54^c^		7.5 ± 0.87^b, e^	10.0 ± 0.81^e^	9.0 ± 0.51^e^	113.6	< 0.001
26.70^ii^	Methyl (13Z)-eicosenoate	1.1 ± 0.06^a^	0.3 ± 0.07^b^	0.3 ± 0.05^b^	0.4 ± 0.17^b^	0.1 ± 0.02^b^	0.5 ± 0.07^b^	0.6 ± 0.01^b^	0.6 ± 0.07^b^	10.13	< 0.001
26.72^i^	Methyl 11-eicosenoate	0.3 ± 0.14^a^	0.8 ± 0.06^a, b^	1.0 ± 0.06^a, b^	0.7 ± 0.16^a^		1.9 ± 0.26^b, c^	2.3 ± 0.30^c^	2.5 ± 0.21^c^	14.44	< 0.001
	Ʃ MUFA	210.6 ± 8.47	91.7 ± 2.71	149.4 ± 4.33	119.7 ± 5.69	27.3 ± 2.65	45.6 ± 4.58	57.0 ± 3.36	52.3 ± 3.60		
25.33^ii^	Methy (9Z,11Z)-octadecadienoate	1.0 ± 0.26^a, b^	0.3 ± 0.06^b, c^	0.4 ± 0.10^b, c^	0.3 ± 0.13^a^	1.5 ± 0.19^c^	0.2 ± 0.13^c^	0.2 ± 0.04^c^	0.2 ± 0.05^c^	8.34	< 0.001
25.40^i^	Methyl (9Z,12Z)-octadecadienoate	302.7 ± 3.14^a^	40.5 ± 1.03^b^	49.8 ± 1.83^b^	17.1 ± 0.91^c^	7.6 ± 0.66^c, d^	13.0 ± 2.41^c, d^	10.8 ± 0.3^c,d^	5.8 ± 0.23^d^	3605	< 0.001
25.55^ii^	Methyl (10E,12Z)-octadecadienoate	50.2 ± 0.14^a^	2.5 ± 0.08^b^	2.9 ± 0.11^b^	0.7 ± 0.39^c^	0.3 ± 0.08^c^				6442	< 0.001
25.57^ii^	Methyl 7,10-octadecadienoate	1.4 ± 0.15^a^	0.1 ± 0.06^a^	0.1 ± 0.07^a^	1.0 ± 0.67^a^					2.16	0.096
25.68^ii^	Methyl (9E,12E)-octadecadienoate	1.3 ± 0.13^a, b^	0.9 ± 0.07^a, b^	0.7 ± 0.10^a, b^	1.4 ± 0.52^a^		0.9 ± 0.13^a, b^			4.663	0.005
25.71^ii^	Methyl (6E,10E, R)-3,7,11,15-tetramethyl-6,10,14-hexadecatrienoate		0.9 ± 0.02^a^	1.3 ± 0.30^a, b^	0.5 ± 0.15^a^	3.2 ± 0.48^b^			12.6 ± 0.76^c^	105.5	< 0.001
25.71^ii^	Methyl (9Z,15Z)-octadecadienoate	46.3 ± 0.26^a^	0.4 ± 0.04^b^	9.4 ± 0.44^c^	0.2 ± 0.05^b^	3.1 ± 0.06^d^	0.3 ± 0.10^b^	0.4 ± 0.04^b^	0.5 ± 0.03^b^	4942	< 0.001
25.94^i^	Methyl 9Z,12Z,15Z-Octadecatrienoate [α-Linolenic acid, (ALA)]	7.9 ± 0.37^a^	9.4 ± 0.61^a^	1.1 ± 0.08^b^	1.3 ± 0.20^b^	1.5 ± 0.39^b^	0.6 ± 0.52^b^	0.5 ± 0.07^b^	0.1 ± 0.04^b^	91.09	< 0.001
26.24^ii^	Methyl (9E,11E)-octadecadienoate	26.3 ± 0.14^a^								7190	< 0.001
26.28^ii^	Methyl (9Z,11E,13E)-octadecatrienoate	9.1 ± 0.16^a^	1.2 ± 0.1^b, c^	1.1 ± 0.1^b, c^	0.6 ± 0.04^b^	1.5 ± 0.21^b, c^	1.0 ± 0.2^b, c^	1.5 ± 0.21^b, c^	1.9 ± 0.19^c^	213.20	< 0.001
26.40^ii^	Methyl 5Z,8Z,11Z,14Z-eicosatetraenoate		1.6 ± 0.1^b, c, d^	2.8 ± 0.32^e^	0.7 ± 0.15^a, c^	0.3 ± 0.05^a, b^	1.5 ± 0.52^c, d^	1.6 ± 0.23^c, d^	1.9 ± 0.11^d, e^	16.43	< 0.001
26.42^ii^	Methyl-(11Z,14Z)-eicosadienoate	8.8 ± 0.14^a^				0.3 ± 0.09^b^				632.8	< 0.001
26.47^i^	Methyl 5Z,8Z,11Z,14Z,17Z-eicosapentaenoate [eicosapentaenoic acid, (EPA)]		25.1 ± 1.23^b^	1.0 ± 0.22^a^	1.4 ± 0.22^a^	0.5 ± 0.12^a^	0.9 ± 0.10^a^	1.3 ± 0.18^a^	0.3 ± 0.04^a^	238.7	< 0.001
26.68^ii^	Methyl (11E,14E)-eicosadienoate	1.4 ± 0.14^a, b^	1.2 ± 0.12^a, b^	1.2 ± 0.99^a^	0.6 ± 0.09^b, c^					20.2	< 0.001
27.98^i^	Methyl 4Z,7Z,10Z,13Z,16Z,19Z-docosahexaenoate [docosahexaenoic acid, DHA]		3.2 ± 0.55^b^	2.0 ± 0.22^b, c^	2.2 ± 0.22^b, c^	0.6 ± 0.14^a, c^	0.4 ± 0.72^a, c^	0.6 ± 0.21^a, c^	0.9 ± 0.44^a, c^	8.311	< 0.001
	Ʃ PUFA	456.41 ± 5.01	87.3 ± 4.01	73.8 ± 3.94	27.9 ± 3.75	20.3 ± 2.48	19.0 ± 2.26	16.9 ± 1.32	24.2 ± 1.89		
	Ʃ n-6 PUFA	463.9 ± 4.29	45.1 ± 1.15	54.6 ± 2.15	19.8 ± 1.25	8.2 ± 0.82	14.0 ± 1.18	10.8 ± 0.33	5.8 ± 0.23		
	Ʃ n-3 PUFA	63.5 ± 1.15	38.7 ± 2.53	14.0 ± 1.04	5.72 ± 0.87	5.7 ± 0.71	3.4 ± 0.85	3.7 ± 0.58	3.1 ± 0.78		
	Ʃ n-6/n-3	7.3	1.2	3.9	3.5	1.4	4.1	2.9	1.9		
	Ʃ ALA + EPA + DHA	7.9 ± 0.37	37.7 ± 2.40	4.1 ± 0.51	4.93 ± 0.64	2.6 ± 0.65	2.0 ± 0.63	2.4 ± 0.46	1.29 ± 0.52		

t_R_ Retention time in mins, ^i^ Compounds identified through co-injection and published library mass spectral databases

ii Compounds tentatively identified by comparison to published library mass spectral databases only; *Mean ± SE (standard error) of triplicate determinations). SFA saturated fatty acids, MUFA monounsaturated fatty acids, PUFA polyunsaturated fatty acids; fatty acid with different lower-case letters are significantly different from each other (ANOVA followed by Student-Neuman-Keul’s (SNK’s) post-hoc test; p < 0.05) for the same fatty acid across oils; blank spaces within the oil concentration data indicate the fatty acid was not detected fatty

The detected fatty acids comprised 35 SFA, 16 MUFA, and 15 PUFA. SFA distribution was as follows: sesame (19), *S. gregaria* L5 (28), *S. icipe* (26), *G. bimaculatus* (28), *S. frugiperda* (17), *B. dorsalis* adult (20), L3 (20), and *C. capitata* (21). MUFA counts were sesame (14), *S. gregaria* L5 (12), *S. icipe* (12), *G. bimaculatus* (12), *S. frugiperda* (6), *B. dorsalis* adult (12), L3 (12), and *C. capitata* (12). PUFA counts were sesame (11), *S. gregaria* L5 (13), *S. icipe* (13), *G. bimaculatus* (13), *S. frugiperda* (11), *B. dorsalis* adult (9), L3 (8), and *C. capitata* (9).

Several fatty acids were detected exclusively in either insect or plant oils. Sixteen SFA were unique to insect oils, whereas one SFA (methyl 4,8-dimethylnonanoate) was exclusive to sesame oil. Two MUFA (methyl (10*Z*)-heptadecenoate and methyl (6*Z*)-octadecenoate) occurred only in insect oils, while two MUFA (methyl (5*Z*)-dodecenoate and methyl 9-heptadecenoate) were found solely in sesame oil. Additionally, four PUFA were detected exclusively in insect oils.

**Fig 2:**
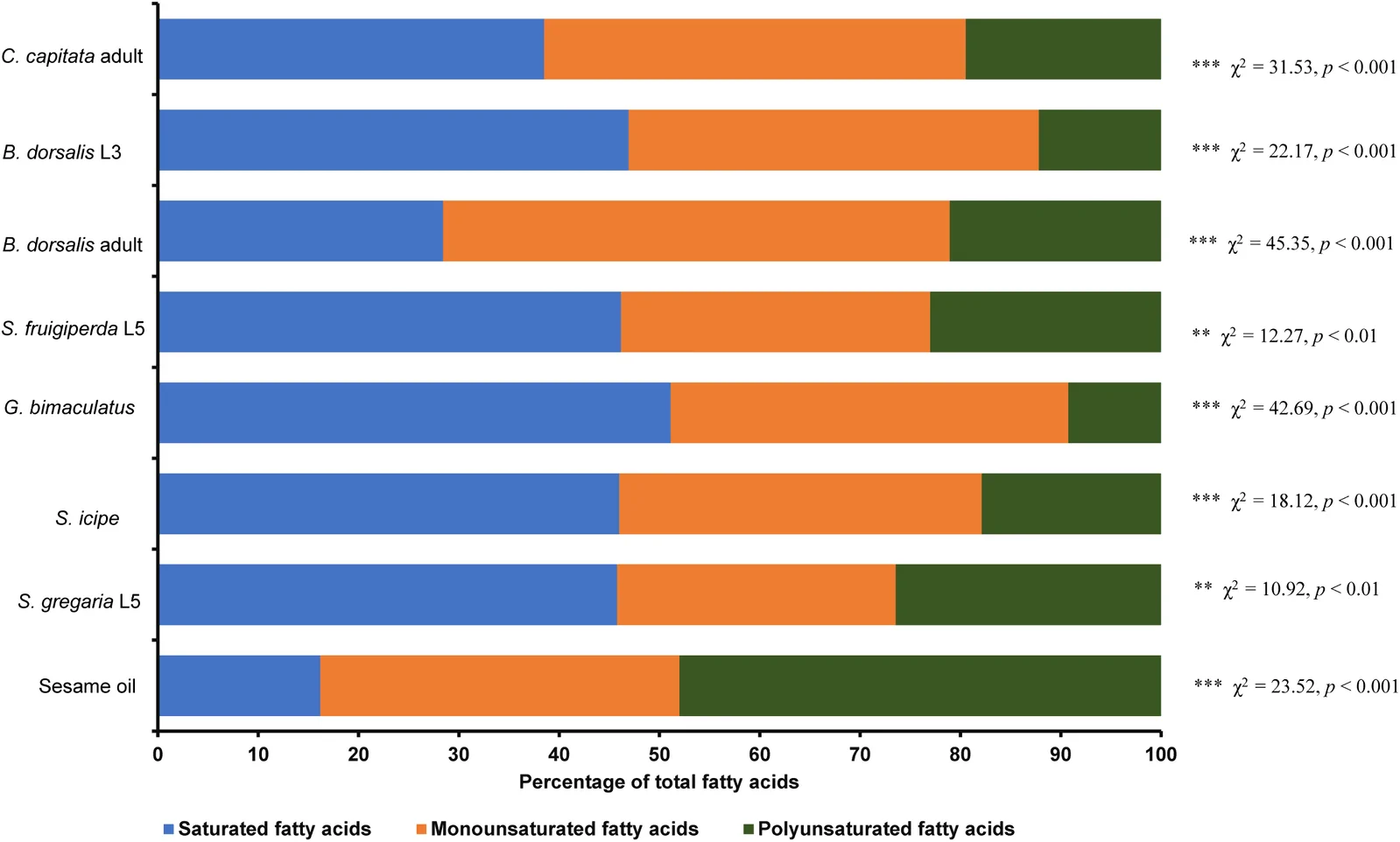
Comparative composition of fatty acid methyl esters in sesame oil and insect oils. Difference in the proportions of SFAs, MUFAs and PUFAs were assessed using the Chi-square test. Statistical significance is indicated as follows ** p < 0.01, *** p < 0.001.

The relative proportions of SFA, MUFA, and PUFA across insect and plant oils are shown (Fig 2). Insect oils displayed generally similar percentages of these fatty acid classes, whereas sesame oil contained a higher proportion of PUFA. Among essential PUFAs, linoleic acid (LA; 18:2 n-6) ranged from 5.8 µg/mg in *C. capitata* adult to 49.8 µg/mg in *S. icipe*. *S. gregaria* L5 contained 40.5 µg/mg of LA, while all orthopteran insect oils (*S. icipe*, *S. gregaria* L5, and *G. bimaculatus*) exceeded 17.1 µg/mg.

ω-3 FA were detected at varying levels across the insect and plant oils (Table 2). ALA was most abundant in *S. gregaria* L5 (9.4 µg/mg), followed by sesame oil (7.9 µg/mg). EPA was highest in *S. gregaria* L5 (25.2 µg/mg), with lower concentrations in *G. bimaculatus* (1.4 µg/mg) and *B. dorsalis* L3 (1.3 µg/mg). (DHA) also peaked in *S. gregaria* L5 (3.2 µg/mg). The ω-6/ω-3 ratios in insect oils ranged from 1.2 to 3.9.

### Vitamin E

**Fig 3:**
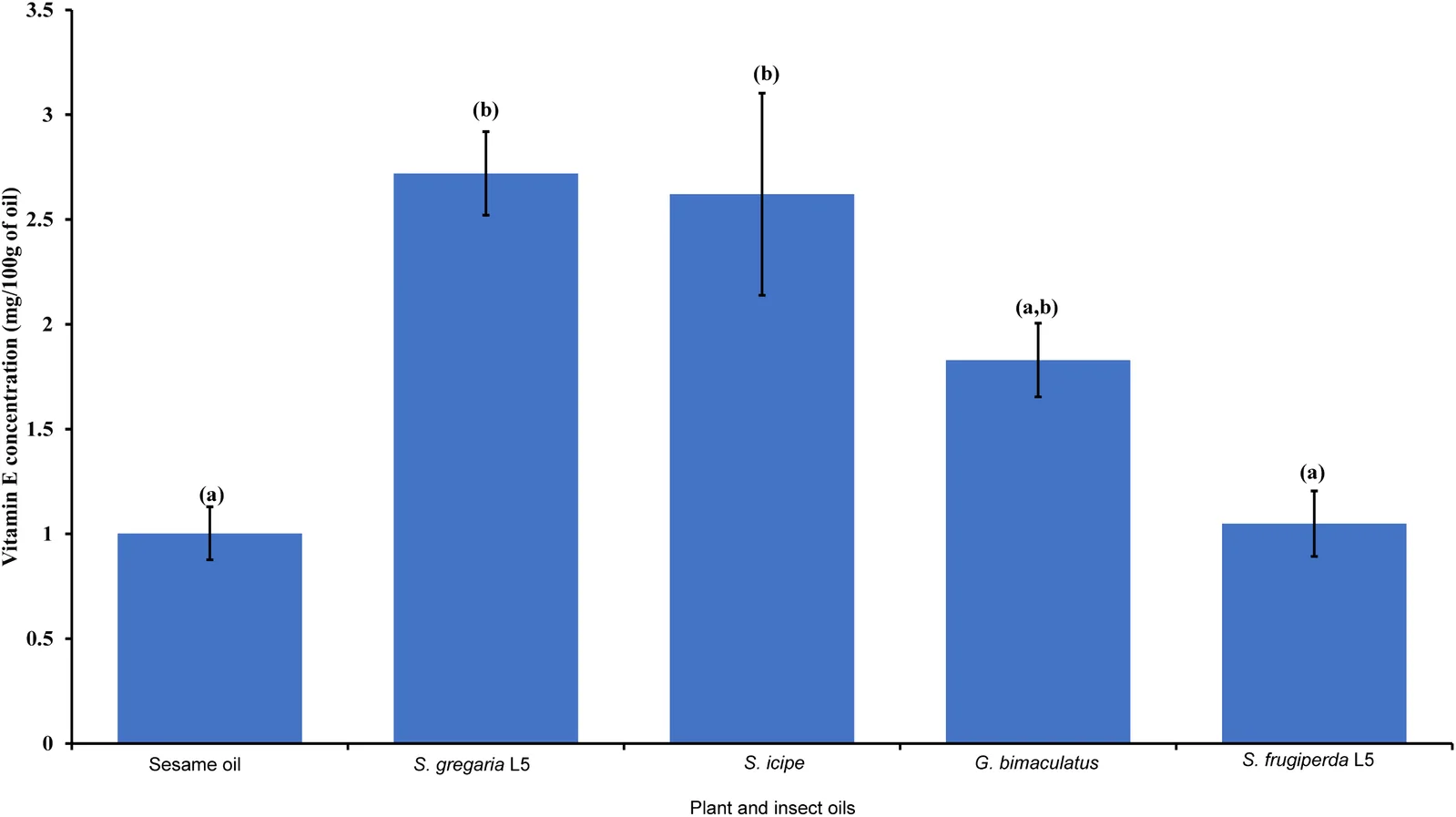
Vitamin E concentration in the different insect and plant oils. On each bar graph, the minimum and maximum values of all the data are represented by the ends of the standard error whiskers. Bar graphs with different lower-case letters are significantly different from each other (ANOVA followed by Student-Neuman-Keul’s (SNK’s) post-hoc test; p < 0.05, n = 3).

Only *α*-tocopherol was detected across all oil samples, and its concentration varied among the different sources. *S*. *gregaria* L5 and*S*. *icipe* recorded higher *α*-tocopherol levels than sesame oil and *S*. *frugiperda* L5 (Fig 3). *G*. *bimaculatus* showed an intermediate *α*-tocopherol concentration (1.83 mg/ 100 g oil), which was numerically higher than that of sesame oil (1.00 mg/100 g oil) but did not differ significantly from either the sesame oil/ *S*. *frugiperda* L5 or the *S*. *gregaria* L5/*S*. *icipe* group.

### Flavonoids

Eight flavonoids were identified with distinct qualitative distributions across the different oils (Table 3). Sesame oil contained three flavonoids; *S*. *gregaria* L5 (5); *S*. *icipe* (3); and *G*. *bimaculatus* (3). Sesame oil uniquely contained high levels of hesperidin and sesamoside, while *S. gregaria* L5 had apigenin and orientin in addition to the three flavonoids common to all insect oils.

**Table 3:** Flavonoids composition (ng/g of oil^*^) in analyzed oils.

t_R_(min)	Flavonoid name	Sesame	*S. gregaria* L5	*S. icipe*	*G. bimaculatus*	*S. frugiperda* L5
6.20	Rutin^i^	22.6 ± 1.25	n. d	n. d	n. d	n. d
6.39	Apigenin^i^	n. d	12.9 ± 1.08	n. d	n. d	n. d
7.98	Quercetin^i^	n. d	38.7 ± 5.47	23.5 ± 2.98	43.6 ± 5.05	33.2 ± 1.03
8.79	Luteolin^i^	n. d	18.9 ± 2.73	5.0 ± 1.09	17.2 ± 6.84	16.2 ± 6.49
9.60	Kaempferol^i^	n. d	62.1 ± 0.56	69.8 ± 6.98	8.7 ± 4.02	39.7 ± 4.87
12.30	Orientin^ii^	n. d	19.1 ± 3.27	n. d	n. d	n. d
12.62	Hesperidin^ii^	50.8 ± 4.14	n. d	n. d	n. d	n. d
12.73	Sesamoside^ii^	67.8 ± 3.03	n. d	n. d	n. d	n. d

t_R,_ Retention time; ^*^Mean ± SE (standard error) of triplicate determinations); ^i^ flavonoids identity confirmed with authentic sample; ^ii^ flavonoids tentatively identified; n.d flavonoid not detected

## Discussion

The extraction method strongly influenced overall oil yields and fatty acid profiles in this study. We employed aqueous extraction, intentionally selected for its safety, affordability, and ability to preserve thermolabile bioactive compounds such as flavonoids and vitamin E [14,26]. This method efficiently recovers polar and amphipathic lipids, including phospholipids, glycolipids, and lysophospholipids, but has limited efficiency for non-polar storage lipids such as triacylglycerols, which are the main carriers of SFAs [29,30]. Therefore, the SFAs reported here primarily reflect the polar lipid fraction rather than the total insect SFA content. Comparisons with previous studies using conventional extraction techniques (e.g., Soxhlet, Folch, or ultrasound-assisted extraction) or solvent-based methods should therefore be interpreted in this context [31]. Future studies incorporating multiple extraction methods would allow comprehensive characterization of the total lipid fraction in edible insects.

Oil yields differ widely among the seven insect species, reflecting variations in developmental stage, species-specific physiology, and extraction method. For example, the desert locust has previously been reported to yield 37 mg/g of oil at adult stage produced nearly twice this amount in the 5^th^ instar using aqueous extraction [14]. Crickets (S*. icipe* and *G. bimaculatus*) exhibited the highest oil yields among Orthoptera, whereas the fruit fly species (*B. dorsalis* and *C. capitata*) yielded considerably less oil, consistent with their smaller body size and lower energetic requirements [32]. The fall armyworm (*S*. *frugiperda*) yielded moderate amounts of oil, highlighting its potential as an abundant and cost-effective biomass source [33,34].

Dietary composition played a critical role in shaping the fatty acid and bioactive compound profiles of insect oils. Desert locusts fed a mixed diet of wheat seedlings, maize leaves, and wheat bran, rich in essential fatty acid precursors and natural antioxidants, exhibited elevated levels of ω-3 FA (ALA, EPA, DHA), α-tocopherol, and flavonoids [35,36]. Crickets reared on complex high-energy diets including wheat bran, soybean, fish offal, pumpkin leaf, and carrot similarly demonstrated enhanced lipid accumulation and bioactive content [37]. In contrast, fall armyworm, fed exclusively on maize leaves, showed a more restricted fatty acid diversity and lower flavonoid content, illustrating the influence of dietary inputs on nutritional quality. Flavonoids such as quercetin, luteolin, kaempferol, and apigenin detected in insect oils likely reflect the presence of these compounds in plant-based diets and may be sequestered or metabolically transformed by insects’ ingestion [38,39]. These findings highlight the potential for targeted dietary manipulation during rearing to optimize the nutritional and functional properties of edible insect oils [40–42].

GC-MS analysis confirmed that edible insect oils contain a nutritionally and functionally significant diversity of SFA, MUFA, and PUFA. *S*. *gregaria* L5, *G*. *bimaculatus*, and *S*. *icipe* showed the highest number of FAMEs, consistent with earlier findings on the biochemical richness of orthopteran oils [14,43]. Conversely, *B*. *dorsalis* and *C*. *capitata* showed fewer FAMEs, like sesame oil. Interestingly, sesame oil displayed a greater diversity of FAMEs than previously documented, suggesting improved analytical sensitivity in this study [44].

Saturated fatty acids were the dominant class (~ 53%), consistent with their roles in insect physiology, including energy storage, membrane integrity, reproduction, and adaptation [45]. Palmitic acid (C16:0), a major SFAs, contributes to cuticle structure and protection [46]. Lauric acid (C12:0), detected in all species, offers antimicrobial protection and supports rapid energy mobilization. Targeted dietary manipulation and optimized rearing could increase the content of nutritionally or industrially valuable SFAs, such as lauric acid [47,48].

In humans, SFAs provide concentrated energy, support thermoregulation, and facilitate the absorption of fat-soluble vitamins, while also enhancing the texture and stability of formulated foods [19,49,50]. SFAs such as palmitic and stearic acid are widely used in cosmetics, soaps, emulsifiers, lubricants, polishes, and emerging biofuel technologies, emphasizing the commercial relevance of insect-derived lipids beyond food applications [51,52].

Unsaturated fatty acids, including MUFA and PUFA, are equally important in insects for maintaining membrane fluidity and for serving as precursors to pheromones and other signaling molecules [50]. In humans, they are known for their cardioprotective and metabolic benefits [1,53,54].

One of the most striking findings was the presence of all three major ω-3 FA, (ALA, EPA, and DHA) in insect oils, usually associated with marine sources (Table 4). *S. gregaria* L5 displayed particularly high EPA (2514 mg/100 g) and DHA (319 mg/100 g), levels comparable to or exceeding several commercial fish species (Table 4).

**Table 4:** Comparison of essential omega-3 fatty acid (mg/100 g) in selected plant seed oils, edible insects and fishes.

	α-linolenic acid (ALA)	Eicosapentaenoic acid (EPA)	Docosahexaenoic acid (DHA)	Ref.
**Plant oils**				
Flaxseeds	22800	0	0	[57]
Chia Seeds	17800	0	0	[58]
Hemp Seeds	8700	0	0	[59]
Walnuts	9100	0	0	[60]
Sesame oil	786	0	0	
**Edible insects**				
*S.gregaria* L5	937	2514	319	
*S. icipe*	114	99	201	
*G. bimaculatus*	129	144	219	
*S. frugiperda*	150	52	55	
*B. dorsalis adult*	65	87	42	
*B. dorsalis* L3	46	130	64	
*C. capitata* adult	14	25	89	
**Fishes**				
Salmon	30	860	1110	[61]
Mackerel	0	1400	1300	[61]
Sardines	0	1000	1200	[61]
Herring	0	700	1000	[61]
Anchovies	0	900	1000	[62]
Tuna	3	280	820	[61]

*G*. *bimaculatus* also contained noteworthy amounts. In contrast, plant oils, such as flaxseed, chia, and sesame, provide ALA but no DHA or EPA. Because human conversion of ALA to EPA and DHA is inefficient (<10%) [55], edible insects offer a more complete ω-3 FA profile, especially for regions with limited access to fish or marine oils. The elevated ω-3 FA levels in *S*. *gregaria* L5 may be influenced by ecological factors such as cannibalism, which warrants further study [56].

These findings position edible insects as credible, environmentally responsible alternatives to marine sources of ω-3 FA, particularly at a time when overfishing, habitat loss, and aquaculture resource demands are intensifying [63]. Their demonstrated health benefits, including cardioprotective, anti-inflammatory, neurocognitive, and dermatological effects [53,54], further support their integration into functional foods and nutraceutical formulations.

The ω-6/ω-3 ratios (1.2–3.9) recorded for insect oils fall within WHO recommendations (<5:1), suggesting favorable dietary potential [64,65]. Current intake recommendations call for 1.1–1.6 g/day ALA and 250–500 mg/day combined EPA + DHA for adults (higher for pregnancy/lactation) [66,67].

Beyond fatty acids, the analyzed insect oils were rich in micronutrients, notably α-tocopherol (vitamin E), which was abundant in several species. Vitamin E acts as a potent antioxidant, protecting cellular membranes from oxidative damage and supporting immune, neurological, and cardiovascular health [11,67]. The high values observed align with earlier reports in diverse insect taxa [13,68], indicating that edible insects can serve as a natural dietary source of vitamin E. The levels of vitamin E in insects are influenced by the rearing diet, with plant-derived substrates rich in tocopherols and other antioxidants contributing to higher concentrations [35–37] For example, *S. gregaria* L5, which fed on a combination of wheat seedlings, maize leaves, and wheat bran, exhibited higher vitamin E content compared to fall armyworm (*S. frugiperda*), reared solely on maize leaves, demonstrating the direct impact of dietary composition on micronutrient accumulation. This suggests that dietary manipulation during insect farming could optimize vitamin E content, enhancing the functional and nutraceutical quality of the oils.

Flavonoids including quercetin, luteolin, kaempferol, and apigenin were detected across species. In insects, flavonoids are typically sequestered from dietary plant sources, as insects generally cannot synthesize these compounds *de novo* [38,39]. The diversity and concentration of flavonoids observed in this study closely reflect the complexity and variety of substrates consumed, with species fed on mixed plant-based diets (e.g., *G. bimaculatus* and *S. icipe*) showing higher flavonoid diversity than species on more restricted diets. These polyphenols contribute antioxidant, anti-inflammatory, and metabolic regulatory benefits, potentially improving oxidative stability of the oils and providing additional health benefits upon consumption [69].

Flavonoids and vitamin E also protect insect tissues from oxidative stress, supporting survival, development, and reproductive fitness [29,70]. For humans, the combination of these bioactives with essential fatty acids enhances the nutritional and therapeutic potential of insect oils, positioning them as promising ingredients for functional foods, nutraceuticals, and cosmeceuticals.

To fully realize the commercial potential of edible insect oils, standardized analytical profiling of vitamin E, flavonoids, other phenolics, alkaloids, and measures of antioxidant capacity and quality indices is essential. Such profiling will enable regulatory compliance, quality assurance, and targeted dietary optimization, ensuring that insect oils meet both nutritional and industrial standards. Future studies should evaluate diet-composition relationships, bioavailability, and stability of these bioactives to strengthen their applicability in food, feed, and pharmaceutical industries.

## Conclusions

Edible insects represent a nutrient-dense and environmentally sustainable source of high-value oils rich in essential ω-3 FA, vitamin E, and diverse flavonoids. The substantial variation in oil yields and fatty acid composition across species and developmental stages underscores opportunities to optimize rearing and extraction strategies. The 5^th^ instar desert locust (*S*. *gregaria*) was particularly notable for providing appreciable levels of ALA, EPA, and DHA, offering a more complete ω-3 FA profile than common plant oils such as sesame. Other species also contributed valuable ω-3 FA, vitamin E, and antioxidant flavonoids, strengthening their potential as functional ingredients. These attributes position insect-derived oils as viable, eco-friendly complementary to conventional plant and marine fats, with broad applications in food, feed, and industrial sectors. Continued research comparing additional plant oils, optimizing production systems, and expanding bioactive profiling will be essential for fully realizing the potential of edible insects within sustainable nutrition and bioeconomy frameworks.
