# Supplementary material for: Omega-3 fatty acids and bioactives in aqueous-extracted oils from seven edible insects
Source: PLoS One. 2026 Aug 26;21(8):e0356807. doi: 10.1371/journal.pone.0356807 (PMC13514048; doi:10.1371/journal.pone.0356807)
Supplement: Supplementary file 1 [file pone.0356807.s001.pdf]

Dear editors:

Attached are my comments about manuscript number: Review PONE-D-26-01997

Article entitled "**Comprehensive insight into insect-derived omega-3 fatty acids and bioactives: Implications for the food and pharmaceutical industry**"

Review

This research article emphasizes the potential significance of insects in the food and pharmaceutical industries, beyond their protein content. The authors quantified and compared oil yields and analyzed the fatty acid profiles, vitamin E content, and flavonoid compositions of seven insect species in relation to commercial sesame oil using GC-MS and LC-MS. This is an important topic, as edible insects offer benefits such as rapid growth and simple production.

Principal impressions

This is a relevant and underexplored topic; however, the authors should make some changes to improve the quality of the manuscript.

1. The short title "***Edible insects as a resource for improved health and nutrition"*** is not appropriate, as this manuscript does not focus on the effect of fatty acids, vitamin E content, and flavonoids on health and nutrition. It should be modified in accordance with the objective and content of the manuscript.
2. The introduction section only mentions omega-3 fatty acids. However, throughout the manuscript, the fatty acid profile, vitamin D content, and flavonoid profile are mentioned, so the latter two should also be described in the introduction.
3. Authors should mention the reason why sesame oil was chosen.
4. Table 2 should be modified; this information should be presented in three separate tables: PUFAs, SFAs, and MUFAs, and the text should be adjusted to the table.
5. Footnote to Table 2. Although the authors point out that the lowercase letters differ significantly from each other, it is unclear whether there is a significant difference between sesame oil and the seven insect species, or whether any of these differ from the others. My suggestion is to present the information as follows: [a] $p = 0.05$ ### vs. #####, [b] $p = 0.02$ #### vs. ####, [c] $p= 0.005$ #### vs

####, as use the legend "*Values are expressed as Mean ± SE (standard error)", and the test used to compare the samples*.

6. Similarly, the legend of Figures 2 and 3 is necessary to describe all the lowercase and tests used to compare samples.
7. Table 3. In this section, the authors report on the statistical significance of almost all flavonoids compared between samples. However, for five flavonoids, only one sample had a detectable amount, so it is not appropriate to perform a statistical analysis in these cases.
8. The discussion section should address the possible effect of the breeding diet on the fatty acid profile, vitamin E content, and flavonoid profile, which is important because this may be causing the difference between the samples analyzed.
