# Supplementary material for: Omega-3 fatty acids and bioactives in aqueous-extracted oils from seven edible insects
Source: PLoS One. 2026 Aug 26;21(8):e0356807. doi: 10.1371/journal.pone.0356807 (PMC13514048; doi:10.1371/journal.pone.0356807)
Supplement: Supplementary file 2 [file pone.0356807.s005.pdf]

Dear editors:

Attached are my comments about manuscript number: Review **PONE-D-26-01997R2**

Article entitled "**Omega-3 fatty acids and bioactives in aqueous-extracted oils from seven edible insects**"

I appreciate the opportunity to review this revised manuscript. After examining this new version, I note that the authors have successfully addressed most of our previous suggestions, which has significantly improved and refined the manuscript. However, there are still some lingering issues regarding Table 2 that require attention before publication.

1. The formatting remains problematic for specific rows. In particular, the fatty acids corresponding to retention times **19.69, 22.23, 24.06, and 26.28** still exhibit overlapping or combined letters.
2. To improve the presentation and readability of the data, the authors should consider rounding the decimal values for specific rows. This applies particularly to the fatty acid values at retention times **30.06, 24.94, and 25.40**, as well as the rows for **Total PUFAs** and **n-3 PUFAs**. Consolidating these numbers via rounding would prevent text from overflowing to the next line within the cells.
