# Supplementary material for: Omega-3 fatty acids and bioactives in aqueous-extracted oils from seven edible insects
Source: PLoS One. 2026 Aug 26;21(8):e0356807. doi: 10.1371/journal.pone.0356807 (PMC13514048; doi:10.1371/journal.pone.0356807)
Supplement: Supplementary file 3 [file pone.0356807.s007.pdf]

Dear Editors:

Please find attached my comments on manuscript no.: Review PONE-D-26-01997R3

Article titled "Omega-3 fatty acids and bioactives in aqueous-extracted oils from seven edible insects"

I appreciate the opportunity to review this revised manuscript. After reviewing this new version, I note that the authors have made the suggested changes. Therefore, I approve the current version and recommend the manuscript for publication.
