# Supplementary material for: Omega-3 fatty acids and bioactives in aqueous-extracted oils from seven edible insects
Source: PLoS One. 2026 Aug 26;21(8):e0356807. doi: 10.1371/journal.pone.0356807 (PMC13514048; doi:10.1371/journal.pone.0356807)
Supplement: Supplementary file 4 [file pone.0356807.s003.docx]

Dear editors:

Attached are my comments about manuscript number: Review PONE-D-26-01997R1

Article entitled “**Omega-3 fatty acids and bioactives in aqueous-extracted oils from seven edible insects**”

I appreciate the opportunity to review this manuscript. After examining this new version, I note that the authors have taken our suggestions into account, which were intended to improve and refine their manuscript. There are a few areas that could be improved.

1. Throughout the manuscript, the authors have used several abbreviations (w-3 FAs, FAs, FAMEs, ALA, EPA, DHA, SFAs, MUFAs, and PUFAs). However, these are not used consistently throughout the manuscript, and in some cases, the authors repeat the full names. For example, in lines 214, 251, 252–255, 313, 317, 328, and 329.

2 In my opinion, there is no need to repeat the ANOVA followed by the Student-Newman-Keuls (SNK) post hoc test in the “Data Analysis” section. The authors could have included the concentrations of individual fatty acids and flavonoids, as well as oil yields and vitamin E concentrations, in the same paragraph.

3 Although the authors have made some changes to Table 2. Fatty acid composition (μ 221 g/mg of insect oil*) analyzed by GC-MS, further modifications are necessary: 1) the text still needs to be adjusted to fit within the cell; in most cases, the letters used to indicate differences between groups and some numbers are carried over to the next line, making it difficult to correctly read the values and differences described in the table. 2) Some values are formatted as superscripts, while some of the letters used are not formatted as superscripts.
