# Supplementary material for: Omega-3 fatty acids and bioactives in aqueous-extracted oils from seven edible insects
Source: PLoS One. 2026 Aug 26;21(8):e0356807. doi: 10.1371/journal.pone.0356807 (PMC13514048; doi:10.1371/journal.pone.0356807)
Supplement: Supplementary file 5 [file pone.0356807.s004.docx]

**Omega-3 fatty acids and bioactives in aqueous-extracted oils from seven edible insects**

**Point-by-Point Response to Reviewers’ Comments**

Dear Prof. Amel Mohamed El Asely

The authors are grateful for the valuable input in the manuscript by reviewers and editors. The important changes made include the following: revision of some parts of the introduction, results, discussion and conclusion sections and the correction of grammatical and syntax errors. Below is the list of point-by-point responses to the reviewer’s comments.

| **Line/Section** | **Comments** | **Authors’ response/action** | **Text revised** |
| --- | --- | --- | --- |
| **Reviewer # 1** | | | |
|  | This study addresses a highly relevant topic concerning alternative protein sources beyond conventional meat and fish. The characterization of species that are already considered edible, including their chemical composition, bioactive compounds, and potential biotechnological applications, is essential to support their broader adoption and valorization within the food system.  Nonetheless are some issues that, in my opinion, should be addressed before the manuscript can be considered suitable for publication | We sincerely thank the reviewer for the positive and insightful evaluation of our manuscript. We greatly appreciate your recognition of the relevance of this study and its contribution to advancing knowledge on alternative food sources, particularly the characterization and valorization of edible insect species. | n/a |
| Title page | 1. Did the authors analyse the total lipid contents using other routine protocols like Bligh and Dyer, Folch or Soxhlet methods as well? In water, only lipids with higher polarity, such as phospholipids, glycolipids, or lysophospholipids, can be partially extracted or dispersed, and even then, with low efficiency. For this reason, the title cannot refer to “Comprehensive insight into insect-derived omega-3 fatty acids“, as the studied oil only corresponded to a fraction of the lipids present in the edible insects. Based on this, the title should be revised in order to reflect what was done here. | We thank the reviewer for this important observation. In this study, we did not use conventional lipid extraction methods such as Bligh and Dyer, Folch, or Soxhlet, this has been done in several other studies within and outside our group. Instead, we used an aqueous extraction approach, which was intentionally selected due to its safety, environmental friendliness, and suitability for preserving thermolabile bioactive compounds.  We acknowledge that aqueous extraction may not recover the total lipid fraction, particularly non-polar lipids, and therefore may not fully represent the complete lipid profile present in the insects. Our aim, however, was to evaluate the composition of the oil fraction obtainable through a simple, scalable, and food-grade extraction method that is more applicable to sustainable processing contexts.  We have revised the title to better reflect the scope of the study and avoid implying a fully comprehensive lipid characterization and now reads ‘***Omega-3 fatty acids and bioactives in aqueous-extracted oils from seven edible insects***. We have also clarified this limitation in the Methods and Discussion sections | Title, Line 91-92 and 286-296 |
| Results | 2. In Table 1, authors refer to *Bactrocera dorsalis* as Fourth instar (L4) but in the Figure 1 legend refer as L3, please revise this | Thank you, this has been revised as *Bactrocera dorsalis* only has up to 3^rd^ instar | Table 1 |
|  | Line 204: remove “fatty acid methyl esters | The word ‘fatty acid methyl esters’ has been deleted | Ln 220 |
|  | Sometimes the authors refer to “standard error of the mean” other to “standard error”. Please harmonize | This has been harmonized to only ‘standard error’ | Ln 214 |
|  | 3. Table 2 should be improved. For example, in the Results section, authors refer to FAME relative content, and these results are not included in the table. | The relative numbers of FAMEs in the Results were calculated based on counts rather than concentrations, and therefore have been removed. We do not wish to include relative values in Table 2. We request to retain Table 2 in its current format, as it provides a clear and concise summary of the total SFA, MUFA, and acids PUFA across the edible insect and sesame oils. | Ln 234-235 |
|  | 4.Results for *B. dorsalis* adult, *B. dorsalis* L3 and *C. capitata* are missing both for vitamin E and Flavonoids. | Thank you for pointing this out. vitamin E and flavonoid analyses were not performed on these oils due to limited sample availability, this statement has been included in the introduction | Ln 95 |
|  | 5. Lines 246-248: according to the statistical data provided in Figure 3, both G. bimaculatus and sesame oil were attributed with letter (a), meaning they have similar contents of vitamin E. Please revise. | The text has been revised accordingly | Ln 264-267 |
|  | 6. The Results section should be significantly improved. The authors mainly focus on the number of identified species, which is not very informative from a food and pharmaceutical industries perspective. Instead, greater emphasis should be placed on the levels (contents) of the compounds, as this information is essential to properly assess the true potential of these oils for the proposed applications. | We thank the reviewer for this insightful comment. We agree that focusing solely on the number of identified compounds may provide limited insight into the nutritional and functional potential of insect oils. However, our results demonstrate meaningful findings, for example, the levels of PUFAs and MUFAs, which are recognized as health-promoting fats, the favorable ω-6/ω-3 ratios (<5:1), and alignment with daily intake recommendations, all of which are discussed in the manuscript.  Our study used aqueous extraction, intentionally selected for its safety, environmental sustainability, and ability to preserve thermolabile bioactive compounds. We acknowledge that this method may not recover the total lipid fraction, particularly non-polar lipids, and therefore may not fully represent the complete lipid profile of the insects. Therefore, the data should be interpreted within this context. | Ln 260-262, 360-395 |
|  | 7. Line 289: The “FAW” abbreviation is not used elsewhere in the manuscript (including the tables) and therefore is unnecessary. | Thank you, the abbreviation ‘FAW’ has been deleted and the discussion section revised | Ln 308 |
|  | 8. The authors state on line 296 that “the extraction method strongly influenced overall yields” and on line 300 that “our use of an aqueous method was intentional, prioritizing safety, affordability, and the preservation of thermolabile bioactives.” These statements are fundamental to the interpretation of the results and should therefore be placed at the very beginning of the Discussion section. Moreover, given that different extraction methodologies can lead to substantially different yields and conclusions, the Discussion requires significant revision to explicitly clarify the basis of comparison. The authors must clearly state whether their results are being compared with those obtained using conventional extraction techniques (e.g., Soxhlet, Folch) or with alternative extraction methods more suitable for food-industry applications. In the absence of this clarification, the Discussion is ambiguous and potentially misleading for readers. 9. Indeed, SFA are typically associated with storage lipids, which are globally composed by triacilglicerol, which are not extracted with water (due to their low polarity). Please revise the sentence based on this. | We thank the reviewer for this important and insightful comment. We fully agree that the extraction method is fundamental to the interpretation of the results. In response, we have substantially revised the opening section of the Discussion to clearly state, at the outset, the influence of the extraction method on both oil yield and lipid composition. | Ln 286-296 |
|  | Indeed, SFA are typically associated with storage lipids, which are globally composed by triacilglicerol, which are not extracted with water (due to their low polarity). Please revise the sentence based on this | Thank you, this has been revised accordingly | Ln 286-293 |
|  | Oil yield results should be expressed in g/100 g, and FAME in mg/100 g which are the unit most commonly adopted | We thank the reviewer for this valuable suggestion. We acknowledge that expressing oil yield in g/100 g and FAME in mg/100 g is a commonly adopted convention. However, we have retained our original units (oil yield in mg/g and FAME in µg/mg) due to the relatively small quantities of oil obtained using the aqueous extraction method.  Converting these values to larger units would involve extrapolation, which may introduce unnecessary scaling errors and reduce precision, particularly given the low extraction efficiency associated with aqueous methods. Reporting the data in the current units allows for more accurate representation of the measured values and minimizes potential rounding or propagation errors. | n/a |
|  | Statistical data on the sum of SFA, MUFA, etc should be included in Table 2 | The statistical summaries for SFA, MUFA, and PUFA are presented immediately after each respective fatty acid group, rather than at the end of the table. The final section is reserved for aggregated metrics, including Σn-6 PUFA, Σn-3 PUFA, the n-6/n-3 ratio, and Σ (ALA + EPA + DHA), which are expressed as fatty acid nutritional indices. This structure was adopted to improve clarity and facilitate interpretation of both individual fatty acid classes and overall nutritional indices, and we therefore wish to retain it in its current form. | Table 2 |
|  | 12. The results for sesame oil should be confirmed as the sum of SFA (185.8 μg/mg), MUFA (416.6 μg/mg) and PUFA (555.8 μg/mg) correspond to 1158 μg/mg, that is 1.158 mg/mg | We thank the reviewer for carefully checking the values and bringing this to our attention. We re-examined the raw data, including peak integration and subsequent calculations and identified that the values for methyl (9Z,12Z)-octadecadienoate and methyl (9Z)-octadecenoate had been inadvertently overestimated during the initial data processing.  After correcting these values, the recalculated total fatty acid content for sesame oil is 854.63 µg/mg.The corresponding values in the table and text have now been updated accordingly. | Table 2, |
|  | 13. Line 304: Please explain in more detail why is relevant a “rich diversity of fatty acids | We agree that the statement “rich diversity of fatty acids” required further clarification and is hanging in the sentence. The richness was in the sense of SFA, MUFA and PUFA that were detected. The sentence has been revised | Ln 353-354 |
|  | 14. Did diets had any influence in fatty acids profile determined in the oils? | Yes, this is now captured in the discussion | Ln 310-322 |
|  | 15. Line 339: replace “Their” by “they” | We thank the reviewer for the suggestion. In this sentence, “Their” is used as a possessive pronoun referring to edible insect oils, which is plural. Replacing it with “they” would alter the grammatical structure and meaning of the sentence, as “they” is a subject pronoun rather than a possessive. Therefore, we propose to retain “Their” in the text. | Ln 389 |
|  |  |  |  |
|  | **Reviewer # 2** | | |
|  | This research article emphasizes the potential significance of insects in the food and pharmaceutical industries, beyond their protein content. The authors quantified and compared oil yields and analyzed the fatty acid profiles, vitamin E content, and flavonoid compositions of seven insect species in relation to commercial sesame oil using GC-MS and LC-MS. This is an important topic, as edible insects offer benefits such as rapid growth and simple production.  This is a relevant and underexplored topic; however, the authors should make some changes to improve the quality of the manuscript. | Thank you | n/a |
| Title page | The short title “***Edible insects as a resource for improved health and nutrition”*** is not appropriate, as this manuscript does not focus on the effect of fatty acids, vitamin E content, and flavonoids on health and nutrition. It should be modified in accordance with the objective and content of the manuscript. | The short title has been modified to align with the objective and content of the manuscript, it now reads ‘***Edible insect oils as sources of omega-3 fatty acids and bioactives*’** | Ln 6 |
| Introduction | The introduction section only mentions omega-3 fatty acids. However, throughout the manuscript, the fatty acid profile, vitamin D content, and flavonoid profile are mentioned, so the latter two should also be described in the introduction. | A paragraph has been added to describe the vit E and flavonoid in edible insect with appropriate references | Ln 65-75 and line 88 |
|  | Authors should mention the reason why sesame oil was chosen. | The paragraph has been revised to show reason why sesame oil was chosen | Line 96-102 |
| Results | Table 2 should be modified; this information should be presented in three  separate tables: PUFAs, SFAs, and MUFAs, and the text should be adjusted to  the table. | We thank the reviewer for the suggestion. However, we would like to retain Table 2 in its current form because it provides a concise summary of SFAs, MUFAs, and PUFAs in a single table, which is clearly illustrated and complemented by Figure 2. Splitting the data into three separate tables may reduce clarity and disrupt the continuity of the comparative analysis. We believe that the current presentation effectively conveys the relevant information while maintaining readability.Text has been adjusted in Table 2 to font 10 | Table 2 |
|  | Footnote to Table 2. Although the authors point out that the lowercase letters differ significantly from each other, it is unclear whether there is a significant difference between sesame oil and the seven insect species, or whether any ofthese differ from the others. My suggestion is to present the information as  follows:  a p = 0.05 ### vs. #####,  bp = 0.02 #### vs. ####,  c p= 0.005 #### vs  ####, as use the legend “***Values are expressed as*** ***Mean ± SE (standard error)”,***  ***and the test used to compare the samples.*** | Thank you, the footnote has been revised to remove ambiguity and the statistics used included. The values are compared across the table 2 for the same fatty acid | Ln 227-232 |
|  | Similarly, the legend of Figures 2 and 3 is necessary to describe all the  lowercase and tests used to compare samples. | The legend has been revised | Line 253-255 and Ln 269 -272 |
|  | Table 3. In this section, the authors report on the statistical significance of  almost all flavonoids compared between samples. However, for five  flavonoids, only one sample had a detectable amount, so it is not appropriate to perform a statistical analysis in these cases. | We thank the reviewer for the suggestion. Statistical analyses have been removed from all flavonoid data to ensure alignment with the comment | Ln 204-205, Table 3. |
| Discussion | The discussion section should address the possible effect of the breeding diet  on the fatty acid profile, vitamin E content, and flavonoid profile, which is important because this may be causing the difference between the samples analyzed. | We agree with the reviewer that diet is a key determinant of the nutritional quality and bioactive composition of edible insects. This aspect has now been incorporated and discussed in the revised manuscript. | Ln 310-322 |
