# Supplementary material for: Omega-3 fatty acids and bioactives in aqueous-extracted oils from seven edible insects
Source: PLoS One. 2026 Aug 26;21(8):e0356807. doi: 10.1371/journal.pone.0356807 (PMC13514048; doi:10.1371/journal.pone.0356807)
Supplement: Supplementary file 6 [file pone.0356807.s006.docx]

**Omega-3 fatty acids and bioactives in aqueous-extracted oils from seven edible insects**

**Point-by-Point Response to Reviewers’ Comments**

Dear Prof. Amel Mohamed El Asely

The authors are grateful for the valuable input in the manuscript by reviewers and editors. The important changes made include the following: revision of some parts of the introduction, results, discussion and conclusion sections and the correction of grammatical and syntax errors. Below is the list of point-by-point responses to the reviewer’s comments.

| **Line/Section** | **Comments** | **Authors’ response/action** | **Text revised** |
| --- | --- | --- | --- |
| **Reviewer # 1** | | | |
| Results | I thank the authors for their responses and for the care taken in revising the manuscript. I believe that the clarifications provided and the revisions implemented have significantly improved the clarity and scientific quality of the work. However, one issue still remains in lines 264–267 concerning the interpretation of the results presented in Figure 3. According to the statistical analysis shown in Figure 3, G. bimaculatus is assigned the letters “a,b”, indicating no significant differences in α-tocopherol content compared with either sesame oil and S. frugiperda (letter “a”) or S. gregaria L5 and S. icipe (letter “b”). Therefore, it is not appropriate to state that G. bimaculatus exhibited higher α-tocopherol levels than sesame oil | We thank the reviewer for this observation. We agree that *G*. *bimaculatus* (a,b) was not significantly different from either sesame oil and *S*. *frugiperda* L5 (a) or *S*. *gregaria* L5 and *S*. *icipe* (b), and therefore our original wording could imply an unsupported significant difference. The text has been revised to clarify that only *S*. *gregaria* L5 and *S*. *icipe* had significantly higher α-tocopherol concentrations than sesame oil, while *G*. *bimaculatus* showed an intermediate concentration that was not significantly different from either group. | 259-264 |
|  | **Reviewer # 2** | | |
| Entire manuscript | I appreciate the opportunity to review this manuscript. After examining this new version, I note that the authors have taken our suggestions into account, which were intended to improve and refine their manuscript. There are a few areas that could be improved.  1. Throughout the manuscript, the authors have used several abbreviations (w-3 FAs, FAs, FAMEs, ALA, EPA, DHA, SFAs, MUFAs, and PUFAs). However, these are not used consistently throughout the manuscript, and in some cases, the authors repeat the full names. For example, in lines 214, 251, 252–255, 313, 317, 328, and 329. | We thank the reviewer for the positive assessment of our revised manuscript and appreciate the constructive feedback aimed at further improving its clarity and quality.  The mentioned abbreviations have been standardized and corrected throught the manuscript | Lines 29, 30,56, 62, 77, 84, 90, 131, 215, 230, 231, 252, 253, 256, 288, 305, 318, 322, 333, 337, 338, 343, 344, 350, 391, 395, 396 |
| Data Analysis | 2. In my opinion, there is no need to repeat the ANOVA followed by the Student-Newman-Keuls (SNK) post hoc test in the “Data Analysis” section. The authors could have included the concentrations of individual fatty acids and flavonoids, as well as oil yields and vitamin E concentrations, in the same paragraph | We thank the reviewer for the suggestion and have revised the Data Analysis section to remove redundancy and improve clarity, while ensuring all reported variables are clearly aligned with the corresponding results tables and text. | Line 192-197 |
| Results | 3. Although the authors have made some changes to Table 2. Fatty acid composition (μ 221 g/mg of insect oil*) analyzed by GC-MS, further modifications are necessary: 1) the text still needs to be adjusted to fit within the cell; in most cases, the letters used to indicate differences between groups and some numbers are carried over to the next line, making it difficult to correctly read the values and differences described in the table. 2) Some values are formatted as superscripts, while some of the letters used are not formatted as superscripts | Thank you the revisions have been effected in the Table 2 | Line 223 |
