# Supplementary material for: Omega-3 fatty acids and bioactives in aqueous-extracted oils from seven edible insects
Source: PLoS One. 2026 Aug 26;21(8):e0356807. doi: 10.1371/journal.pone.0356807 (PMC13514048; doi:10.1371/journal.pone.0356807)
Supplement: Supplementary file 7 [file pone.0356807.s008.docx]

**Omega-3 fatty acids and bioactives in aqueous-extracted oils from seven edible insects**

**Point-by-Point Response to Reviewers’ Comments**

Dear Prof.

The authors are grateful for the valuable input in the manuscript by reviewers and editors. The important changes have been made in Table 2.

| **Line/Section** | **Comments** | **Authors’ response/action** | **Text revised** |
| --- | --- | --- | --- |
|  | **Reviewer # 2** | | |
| Table 2 | I appreciate the opportunity to review this revised manuscript. After examining this new version, I note that the authors have successfully addressed most of our previous suggestions, which has significantly improved and refined the manuscript. However, there are still some lingering issues regarding Table 2 that require attention before publication. | We thank the reviewer for the positive assessment of our revised manuscript and for recognizing the improvements made; we have carefully addressed the remaining concerns regarding Table 2 in the revised version. | Table 2 |
|  | 1. The formatting remains problematic for specific rows. In particular, the fatty acids corresponding to retention times 19.69, 22.23, 24.06, and 26.28 still exhibit overlapping or combined letters. | This has now been addressed by deleting the column titled ω-n (∆n), which was of lesser importance to the dataset, thereby allowing the remaining columns to be readjusted and improving the overall readability of the table. |  |
|  | 2.To improve the presentation and readability of the data, the authors should consider rounding the decimal values for specific rows. This applies particularly to the fatty acid values at retention times 30.06, 24.94, and 25.40, as well as the rows for Total PUFAs and n-3 PUFAs. Consolidating these numbers via rounding would prevent text from overflowing to the next line within the cells. | This has now been addressed by deleting the column titled ω-n (∆n), which was of lesser importance to the dataset, thereby allowing the remaining columns to be readjusted and improving the overall readability of the table. |  |
