# Supplementary material for: Omega-3 fatty acids and bioactives in aqueous-extracted oils from seven edible insects
Source: PLoS One. 2026 Aug 26;21(8):e0356807. doi: 10.1371/journal.pone.0356807 (PMC13514048; doi:10.1371/journal.pone.0356807)
Supplement: Peer review history

# Peer review: Decision Letter 0

**DOI:** 10.1371/journal.pone.0356807.r001

**Authors:** Amel El Asely

23 Feb 2026

Dear Dr. Cheseto,

Please submit your revised manuscript by Apr 09 2026 11:59PM. If you will need more time than this to complete your revisions, please reply to this message or contact the journal office at plosone@plos.org. When you're ready to submit your revision, log on to https://www.editorialmanager.com/pone/ and select the 'Submissions Needing Revision' folder to locate your manuscript file.

- A letter that responds to each point raised by the academic editor and reviewer(s). You should upload this letter as a separate file labeled 'Response to Reviewers'.
- A marked-up copy of your manuscript that highlights changes made to the original version. You should upload this as a separate file labeled 'Revised Manuscript with Track Changes'.
- An unmarked version of your revised paper without tracked changes. You should upload this as a separate file labeled 'Manuscript'.

If you would like to make changes to your financial disclosure, please include your updated statement in your cover letter. Guidelines for resubmitting your figure files are available below the reviewer comments at the end of this letter.

If applicable, we recommend that you deposit your laboratory protocols in protocols.io to enhance the reproducibility of your results. Protocols.io assigns your protocol its own identifier (DOI) so that it can be cited independently in the future. For instructions see: https://journals.plos.org/plosone/s/submission-guidelines#loc-laboratory-protocols. Additionally, PLOS ONE offers an option for publishing peer-reviewed Lab Protocol articles, which describe protocols hosted on protocols.io. Read more information on sharing protocols at https://plos.org/protocols?utm_medium=editorial-email&utm_source=authorletters&utm_campaign=protocols.

We look forward to receiving your revised manuscript.

Kind regards,

Amel Mohamed El Asely

Academic Editor

PLOS One

Journal Requirements:

When submitting your revision, we need you to address these additional requirements.

1. Please ensure that your manuscript meets PLOS ONE's style requirements, including those for file naming. The PLOS ONE style templates can be found at

https://journals.plos.org/plosone/s/file?id=wjVg/PLOSOne_formatting_sample_main_body.pdf and

https://journals.plos.org/plosone/s/file?id=ba62/PLOSOne_formatting_sample_title_authors_affiliations.pdf

2.  Please note that PLOS One has specific guidelines on code sharing for submissions in which author-generated code underpins the findings in the manuscript. In these cases, we expect all author-generated code to be made available without restrictions upon publication of the work. Please review our guidelines at https://journals.plos.org/plosone/s/materials-and-software-sharing#loc-sharing-code and ensure that your code is shared in a way that follows best practice and facilitates reproducibility and reuse.

3. Thank you for stating the following financial disclosure:

“CMT

Bill & Melinda Gates Foundation (INV-032416); European Commission (NESTLER project: 101060762 and INNOECOFOOD project: 101136739); the Curt Bergfors Foundation Food Planet Prize Award; the Swedish International Development Cooperation Agency (Sida); the Swiss Agency for Development and Cooperation (SDC); the Australian Centre for International Agricultural Research (ACIAR); the Government of Norway; the German Federal Ministry for Economic Cooperation and Development (BMZ); and the Government of the Republic of Kenya”

Please state what role the funders took in the study. If the funders had no role, please state: "The funders had no role in study design, data collection and analysis, decision to publish, or preparation of the manuscript."

If this statement is not correct you must amend it as needed.

Please include this amended Role of Funder statement in your cover letter; we will change the online submission form on your behalf.

4. We note that your Data Availability Statement is currently as follows: If the data are all contained within the manuscript and/or Supporting Information files, enter the following: All relevant data are within the manuscript and its Supporting Information files.

Please confirm at this time whether or not your submission contains all raw data required to replicate the results of your study. Authors must share the “minimal data set” for their submission. PLOS defines the minimal data set to consist of the data required to replicate all study findings reported in the article, as well as related metadata and methods (https://journals.plos.org/plosone/s/data-availability#loc-minimal-data-set-definition).

For example, authors should submit the following data:

- The values behind the means, standard deviations and other measures reported;

- The values used to build graphs;

- The points extracted from images for analysis.

Authors do not need to submit their entire data set if only a portion of the data was used in the reported study.

If your submission does not contain these data, please either upload them as Supporting Information files or deposit them to a stable, public repository and provide us with the relevant URLs, DOIs, or accession numbers. For a list of recommended repositories, please see https://journals.plos.org/plosone/s/recommended-repositories.

If there are ethical or legal restrictions on sharing a de-identified data set, please explain them in detail (e.g., data contain potentially sensitive information, data are owned by a third-party organization, etc.) and who has imposed them (e.g., an ethics committee). Please also provide contact information for a data access committee, ethics committee, or other institutional body to which data requests may be sent. If data are owned by a third party, please indicate how others may request data access.

5. When completing the data availability statement of the submission form, you indicated that you will make your data available on acceptance. We strongly recommend all authors decide on a data sharing plan before acceptance, as the process can be lengthy and hold up publication timelines. Please note that, though access restrictions are acceptable now, your entire data will need to be made freely accessible if your manuscript is accepted for publication. This policy applies to all data except where public deposition would breach compliance with the protocol approved by your research ethics board. If you are unable to adhere to our open data policy, please kindly revise your statement to explain your reasoning and we will seek the editor's input on an exemption. Please be assured that, once you have provided your new statement, the assessment of your exemption will not hold up the peer review process.

6. If the reviewer comments include a recommendation to cite specific previously published works, please review and evaluate these publications to determine whether they are relevant and should be cited. There is no requirement to cite these works unless the editor has indicated otherwise.

[Note: HTML markup is below. Please do not edit.]

Reviewers' comments:

Reviewer's Responses to Questions

**Comments to the Author**

1. Is the manuscript technically sound, and do the data support the conclusions?

Reviewer #1: Yes

Reviewer #2: Yes

**********

2. Has the statistical analysis been performed appropriately and rigorously?

Reviewer #1: Yes

Reviewer #2: Yes

**********

3. Have the authors made all data underlying the findings in their manuscript fully available?

The PLOS Data policy

Reviewer #1: Yes

Reviewer #2: Yes

**********

4. Is the manuscript presented in an intelligible fashion and written in standard English?

Reviewer #1: Yes

Reviewer #2: Yes

**********

Reviewer #1: This study addresses a highly relevant topic concerning alternative protein sources beyond conventional meat and fish. The characterization of species that are already considered edible, including their chemical composition, bioactive compounds, and potential biotechnological applications, is essential to support their broader adoption and valorization within the food system.

Nonetheless are some issues that, in my opinion, should be addressed before the manuscript can be considered suitable for publication.

1. Did the authors analyse the total lipid contents using other routine protocols like Bligh and Dyer, Folch or Soxhlet methods as well? In water, only lipids with higher polarity, such as phospholipids, glycolipids, or lysophospholipids, can be partially extracted or dispersed, and even then, with low efficiency. For this reason, the title cannot refer to “Comprehensive insight into insect-derived omega-3 fatty acids“, as the studied oil only corresponded to a fraction of the lipids present in the edible insects. Based on this, the title should be revised in order to reflect what was done here.

2. In Table 1, authors refer to Bactrocera dorsalis as Fourth instar (L4) but in the Figure 1 legend refer as L3, please revise this.

1. Line 204: remove “fatty acid methyl esters”.

2. Sometimes the authors refer to “standard error of the mean” other to “standard error”. Please harmonize.

3. Table 2 should be improved. For example, in the Results section, authors refer to FAME relative content, and these results are not included in the table.

4. Results for B. dorsalis adult, B. dorsalis L3 and C. capitate are missing both for vitamin E and Flavonoids.

5. Lines 246-248: according to the statistical data provided in Figure 3, both G. bimaculatus and sesame oil were attributed with letter (a), meaning they have similar contents of vitamin E. Please revise.

6. The Results section should be significantly improved. The authors mainly focus on the number of identified species, which is not very informative from a food and pharmaceutical industries perspective. Instead, greater emphasis should be placed on the levels (contents) of the compounds, as this information is essential to properly assess the true potential of these oils for the proposed applications.

7. Line 289: The “FAW” abbreviation is not used elsewhere in the manuscript (including the tables) and therefore is unnecessary.

8. The authors state on line 296 that “the extraction method strongly influenced overall yields” and on line 300 that “our use of an aqueous method was intentional, prioritizing safety, affordability, and the preservation of thermolabile bioactives.” These statements are fundamental to the interpretation of the results and should therefore be placed at the very beginning of the Discussion section. Moreover, given that different extraction methodologies can lead to substantially different yields and conclusions, the Discussion requires significant revision to explicitly clarify the basis of comparison. The authors must clearly state whether their results are being compared with those obtained using conventional extraction techniques (e.g., Soxhlet, Folch) or with alternative extraction methods more suitable for food-industry applications. In the absence of this clarification, the Discussion is ambiguous and potentially misleading for readers.

9. Indeed, SFA are typically associated with storage lipids, which are globally composed by triacilglicerol, which are not extracted with water (due to their low polarity). Please revise the sentence based on this.

10. Oil yield results should be expressed in g/100 g, and FAME in mg/100 g which are the unit most commonly adopted.

11. Statistical data on the sum of SFA, MUFA, etc should be included in Table 2.

12. The results for sesame oil should be confirmed as the sum of SFA (185.8 �g/mg), MUFA (416.6 �g/mg) and PUFA (555.8 �g/mg) correspond to 1158 �g/mg, that is 1.158 mg/mg…

13. Line 304: Please explain in more detail why is relevant a “rich diversity of fatty acids”.

14. Did diets had any influence in fatty acids profile determined in the oils?

15. Line 339: replace “Their” by “they”

16. The discussion of vitamin E and flavonoids is scarce and should be improved.

Reviewer #2: Dear editors:

Attached are my comments about manuscript number: Review PONE-D-26-01997

Article entitled “Comprehensive insight into insect-derived omega-3 fatty acids and bioactives: Implications for the food and pharmaceutical industry”

Review

This research article emphasizes the potential significance of insects in the food and pharmaceutical industries, beyond their protein content. The authors quantified and compared oil yields and analyzed the fatty acid profiles, vitamin E content, and flavonoid compositions of seven insect species in relation to commercial sesame oil using GC-MS and LC-MS. This is an important topic, as edible insects offer benefits such as rapid growth and simple production.

Principal impressions

This is a relevant and underexplored topic; however, the authors should make some changes to improve the quality of the manuscript.

1. The short title “Edible insects as a resource for improved health and nutrition” is not appropriate, as this manuscript does not focus on the effect of fatty acids, vitamin E content, and flavonoids on health and nutrition. It should be modified in accordance with the objective and content of the manuscript.

2. The introduction section only mentions omega-3 fatty acids. However, throughout the manuscript, the fatty acid profile, vitamin D content, and flavonoid profile are mentioned, so the latter two should also be described in the introduction.

3. Authors should mention the reason why sesame oil was chosen.

4. Table 2 should be modified; this information should be presented in three separate tables: PUFAs, SFAs, and MUFAs, and the text should be adjusted to the table.

5. Footnote to Table 2. Although the authors point out that the lowercase letters differ significantly from each other, it is unclear whether there is a significant difference between sesame oil and the seven insect species, or whether any of these differ from the others. My suggestion is to present the information as follows: a p = 0.05 ### vs. #####, b p = 0.02 #### vs. ####, c p= 0.005 #### vs ####, as use the legend “Values are expressed as Mean ± SE (standard error)”, and the test used to compare the samples.

6. Similarly, the legend of Figures 2 and 3 is necessary to describe all the lowercase and tests used to compare samples.

7. Table 3. In this section, the authors report on the statistical significance of almost all flavonoids compared between samples. However, for five flavonoids, only one sample had a detectable amount, so it is not appropriate to perform a statistical analysis in these cases.

8. The discussion section should address the possible effect of the breeding diet on the fatty acid profile, vitamin E content, and flavonoid profile, which is important because this may be causing the difference between the samples analyzed.

**********

what does this mean?). If published, this will include your full peer review and any attached files.

If you choose “no”, your identity will remain anonymous but your review may still be made public.

**Do you want your identity to be public for this peer review?**  For information about this choice, including consent withdrawal, please see our Privacy Policy

Reviewer #1: No

Reviewer #2: **Yes:** Salvador Atilano Miguel

**********

[NOTE: If reviewer comments were submitted as an attachment file, they will be attached to this email and accessible via the submission site. Please log into your account, locate the manuscript record, and check for the action link "View Attachments". If this link does not appear, there are no attachment files.]

To ensure your figures meet our technical requirements, please review our figure guidelines: https://journals.plos.org/plosone/s/figures

You may also use PLOS’s free figure tool, NAAS, to help you prepare publication quality figures: https://journals.plos.org/plosone/s/figures#loc-tools-for-figure-preparation.

NAAS will assess whether your figures meet our technical requirements by comparing each figure against our figure specifications.

Attachment Submitted filename: Review PONE-D-26-01997.pdf

# Author comment: Author response to Decision Letter 1

**DOI:** 10.1371/journal.pone.0356807.r002

13 Apr 2026

Omega-3 fatty acids and bioactives in aqueous-extracted oils from seven edible insects

Point-by-Point Response to Reviewers’ Comments

Dear Prof. Amel Mohamed El Asely

The authors are grateful for the valuable input in the manuscript by reviewers and editors. The important changes made include the following: revision of some parts of the introduction, results, discussion and conclusion sections and the correction of grammatical and syntax errors. Below is the list of point-by-point responses to the reviewer’s comments.

Line/Section Comments Authors’ response/action Text revised

Reviewer # 1

This study addresses a highly relevant topic concerning alternative protein sources beyond conventional meat and fish. The characterization of species that are already considered edible, including their chemical composition, bioactive compounds, and potential biotechnological applications, is essential to support their broader adoption and valorization within the food system.

Nonetheless are some issues that, in my opinion, should be addressed before the manuscript can be considered suitable for publication We sincerely thank the reviewer for the positive and insightful evaluation of our manuscript. We greatly appreciate your recognition of the relevance of this study and its contribution to advancing knowledge on alternative food sources, particularly the characterization and valorization of edible insect species. n/a

Title page 1. Did the authors analyse the total lipid contents using other routine protocols like Bligh and Dyer, Folch or Soxhlet methods as well? In water, only lipids with higher polarity, such as phospholipids, glycolipids, or lysophospholipids, can be partially extracted or dispersed, and even then, with low efficiency. For this reason, the title cannot refer to “Comprehensive insight into insect-derived omega-3 fatty acids“, as the studied oil only corresponded to a fraction of the lipids present in the edible insects. Based on this, the title should be revised in order to reflect what was done here. We thank the reviewer for this important observation. In this study, we did not use conventional lipid extraction methods such as Bligh and Dyer, Folch, or Soxhlet, this has been done in several other studies within and outside our group. Instead, we used an aqueous extraction approach, which was intentionally selected due to its safety, environmental friendliness, and suitability for preserving thermolabile bioactive compounds.

We acknowledge that aqueous extraction may not recover the total lipid fraction, particularly non-polar lipids, and therefore may not fully represent the complete lipid profile present in the insects. Our aim, however, was to evaluate the composition of the oil fraction obtainable through a simple, scalable, and food-grade extraction method that is more applicable to sustainable processing contexts.

We have revised the title to better reflect the scope of the study and avoid implying a fully comprehensive lipid characterization and now reads ‘Omega-3 fatty acids and bioactives in aqueous-extracted oils from seven edible insects. We have also clarified this limitation in the Methods and Discussion sections Title, Line 91-92 and 286-296

Results 2. In Table 1, authors refer to Bactrocera dorsalis as Fourth instar (L4) but in the Figure 1 legend refer as L3, please revise this Thank you, this has been revised as Bactrocera dorsalis only has up to 3rd instar Table 1

Line 204: remove “fatty acid methyl esters The word ‘fatty acid methyl esters’ has been deleted Ln 220

Sometimes the authors refer to “standard error of the mean” other to “standard error”. Please harmonize This has been harmonized to only ‘standard error’ Ln 214

3. Table 2 should be improved. For example, in the Results section, authors refer to FAME relative content, and these results are not included in the table.

The relative numbers of FAMEs in the Results were calculated based on counts rather than concentrations, and therefore have been removed. We do not wish to include relative values in Table 2. We request to retain Table 2 in its current format, as it provides a clear and concise summary of the total SFA, MUFA, and acids PUFA across the edible insect and sesame oils. Ln 234-235

4.Results for B. dorsalis adult, B. dorsalis L3 and C. capitata are missing both for vitamin E and Flavonoids. Thank you for pointing this out. vitamin E and flavonoid analyses were not performed on these oils due to limited sample availability, this statement has been included in the introduction Ln 95

5. Lines 246-248: according to the statistical data provided in Figure 3, both G. bimaculatus and sesame oil were attributed with letter (a), meaning they have similar contents of vitamin E. Please revise. The text has been revised accordingly Ln 264-267

6. The Results section should be significantly improved. The authors mainly focus on the number of identified species, which is not very informative from a food and pharmaceutical industries perspective. Instead, greater emphasis should be placed on the levels (contents) of the compounds, as this information is essential to properly assess the true potential of these oils for the proposed applications. We thank the reviewer for this insightful comment. We agree that focusing solely on the number of identified compounds may provide limited insight into the nutritional and functional potential of insect oils. However, our results demonstrate meaningful findings, for example, the levels of PUFAs and MUFAs, which are recognized as health-promoting fats, the favorable ω-6/ω-3 ratios (<5:1), and alignment with daily intake recommendations, all of which are discussed in the manuscript.

Our study used aqueous extraction, intentionally selected for its safety, environmental sustainability, and ability to preserve thermolabile bioactive compounds. We acknowledge that this method may not recover the total lipid fraction, particularly non-polar lipids, and therefore may not fully represent the complete lipid profile of the insects. Therefore, the data should be interpreted within this context. Ln 260-262, 360-395

7. Line 289: The “FAW” abbreviation is not used elsewhere in the manuscript (including the tables) and therefore is unnecessary.

Thank you, the abbreviation ‘FAW’ has been deleted and the discussion section revised Ln 308

8. The authors state on line 296 that “the extraction method strongly influenced overall yields” and on line 300 that “our use of an aqueous method was intentional, prioritizing safety, affordability, and the preservation of thermolabile bioactives.” These statements are fundamental to the interpretation of the results and should therefore be placed at the very beginning of the Discussion section. Moreover, given that different extraction methodologies can lead to substantially different yields and conclusions, the Discussion requires significant revision to explicitly clarify the basis of comparison. The authors must clearly state whether their results are being compared with those obtained using conventional extraction techniques (e.g., Soxhlet, Folch) or with alternative extraction methods more suitable for food-industry applications. In the absence of this clarification, the Discussion is ambiguous and potentially misleading for readers.

9. Indeed, SFA are typically associated with storage lipids, which are globally composed by triacilglicerol, which are not extracted with water (due to their low polarity). Please revise the sentence based on this. We thank the reviewer for this important and insightful comment. We fully agree that the extraction method is fundamental to the interpretation of the results. In response, we have substantially revised the opening section of the Discussion to clearly state, at the outset, the influence of the extraction method on both oil yield and lipid composition.

Ln 286-296

Indeed, SFA are typically associated with storage lipids, which are globally composed by triacilglicerol, which are not extracted with water (due to their low polarity). Please revise the sentence based on this Thank you, this has been revised accordingly Ln 286-293

Oil yield results should be expressed in g/100 g, and FAME in mg/100 g which are the unit most commonly adopted We thank the reviewer for this valuable suggestion. We acknowledge that expressing oil yield in g/100 g and FAME in mg/100 g is a commonly adopted convention. However, we have retained our original units (oil yield in mg/g and FAME in µg/mg) due to the relatively small quantities of oil obtained using the aqueous extraction method.

Converting these values to larger units would involve extrapolation, which may introduce unnecessary scaling errors and reduce precision, particularly given the low extraction efficiency associated with aqueous methods. Reporting the data in the current units allows for more accurate representation of the measured values and minimizes potential rounding or propagation errors.

n/a

Statistical data on the sum of SFA, MUFA, etc should be included in Table 2 The statistical summaries for SFA, MUFA, and PUFA are presented immediately after each respective fatty acid group, rather than at the end of the table. The final section is reserved for aggregated metrics, including Σn-6 PUFA, Σn-3 PUFA, the n-6/n-3 ratio, and Σ (ALA + EPA + DHA), which are expressed as fatty acid nutritional indices. This structure was adopted to improve clarity and facilitate interpretation of both individual fatty acid classes and overall nutritional indices, and we therefore wish to retain it in its current form. Table 2

12. The results for sesame oil should be confirmed as the sum of SFA (185.8 �g/mg), MUFA (416.6 �g/mg) and PUFA (555.8 �g/mg) correspond to 1158 �g/mg, that is 1.158 mg/mg We thank the reviewer for carefully checking the values and bringing this to our attention. We re-examined the raw data, including peak integration and subsequent calculations and identified that the values for methyl (9Z,12Z)-octadecadienoate and methyl (9Z)-octadecenoate had been inadvertently overestimated during the initial data processing.

After correcting these values, the recalculated total fatty acid content for sesame oil is 854.63 µg/mg.The corresponding values in the table and text have now been updated accordingly. Table 2,

13. Line 304: Please explain in more detail why is relevant a “rich diversity of fatty acids We agree that the statement “rich diversity of fatty acids” required further clarification and is hanging in the sentence. The richness was in the sense of SFA, MUFA and PUFA that were detected. The sentence has been revised Ln 353-354

14. Did diets had any influence in fatty acids profile determined in the oils? Yes, this is now captured in the discussion Ln 310-322

15. Line 339: replace “Their” by “they” We thank the reviewer for the suggestion. In this sentence, “Their” is used as a possessive pronoun referring to edible insect oils, which is plural. Replacing it with “they” would alter the grammatical structure and meaning of the sentence, as “they” is a subject pronoun rather than a possessive. Therefore, we propose to retain “Their” in the text. Ln 389

Reviewer # 2

This research article emphasizes the potential significance of insects in the food and pharmaceutical industries, beyond their protein content. The authors quantified and compared oil yields and analyzed the fatty acid profiles, vitamin E content, and flavonoid compositions of seven insect species in relation to commercial sesame oil using GC-MS and LC-MS. This is an important topic, as edible insects offer benefits such as rapid growth and simple production.

This is a relevant and underexplored topic; however, the authors should make some changes to improve the quality of the manuscript. Thank you n/a

Title page The short title “Edible insects as a resource for improved health and nutrition” is not appropriate, as this manuscript does not focus on the effect of fatty acids, vitamin E content, and flavonoids on health and nutrition. It should be modified in accordance with the objective and content of the manuscript. The short title has been modified to align with the objective and content of the manuscript, it now reads ‘Edible insect oils as sources of omega-3 fatty acids and bioactives’ Ln 6

Introduction The introduction section only mentions omega-3 fatty acids. However, throughout the manuscript, the fatty acid profile, vitamin D content, and flavonoid profile are mentioned, so the latter two should also be described in the introduction. A paragraph has been added to describe the vit E and flavonoid in edible insect with appropriate references Ln 65-75 and line 88

Authors should mention the reason why sesame oil was chosen. The paragraph has been revised to show reason why sesame oil was chosen Line 96-102

Results Table 2 should be modified; this information should be presented in three

separate tables: PUFAs, SFAs, and MUFAs, and the text should be adjusted to

the table. We thank the reviewer for the suggestion. However, we would like to retain Table 2 in its current form because it provides a concise summary of SFAs, MUFAs, and PUFAs in a single table, which is clearly illustrated and complemented by Figure 2. Splitting the data into three separate tables may reduce clarity and disrupt the continuity of the comparative analysis. We believe that the current presentation effectively conveys the relevant information while maintaining readability. Table 2

Footnote to Table 2. Although the authors point out that the lowercase letters differ significantly from each other, it is unclear whether there is a significant difference between sesame oil and the seven insect species, or whether any ofthese differ from the others. My suggestion is to present the information as

follows:

a p = 0.05 ### vs. #####,

bp = 0.02 #### vs. ####,

c p= 0.005 #### vs

####, as use the legend “Values are expressed as Mean ± SE (standard error)”,

and the test used to compare the samples. Thank you, the footnote has been revised to remove ambiguity and the statistics used included. The values are compared across the table 2 for the same fatty acid Ln 227-232

Similarly, the legend of Figures 2 and 3 is necessary to describe all the

lowercase and tests used to compare samples. The legend has been revised Line 253-255 and Ln 269 -272

Table 3. In this section, the authors report on the statistical significance of

almost all flavonoids compared between samples. However, for five

flavonoids, only one sample had a detectable amount, so it is not appropriate to perform a statistical analysis in these cases. We thank the reviewer for the suggestion. Statistical analyses have been removed from all flavonoid data to ensure alignment with the comment Ln 204-205, Table 3.

Discussion The discussion section should address the possible effect of the breeding diet

on the fatty acid profile, vitamin E content, and flavonoid profile, which is important because this may be causing the difference between the samples analyzed. We agree with the reviewer that diet is a key determinant of the nutritional quality and bioactive composition of edible insects. This aspect has now been incorporated and discussed in the revised manuscript. Ln 310-322

Attachment Submitted filename: Response to Reviewers.docx

# Peer review: Decision Letter 1

**DOI:** 10.1371/journal.pone.0356807.r003

**Authors:** Amel El Asely

21 May 2026

Dear Dr. Cheseto,

Thank you for submitting your manuscript to PLOS ONE. After careful consideration, we feel that it has merit but does not fully meet PLOS ONE’s publication criteria as it currently stands. Therefore, we invite you to submit a revised version of the manuscript that addresses the points raised during the review process.

Please submit your revised manuscript by Jul 05 2026 11:59PM. If you will need more time than this to complete your revisions, please reply to this message or contact the journal office at plosone@plos.org. When you're ready to submit your revision, log on to https://www.editorialmanager.com/pone/ and select the 'Submissions Needing Revision' folder to locate your manuscript file.

- A letter that responds to each point raised by the academic editor and reviewer(s). You should upload this letter as a separate file labeled 'Response to Reviewers'.
- A marked-up copy of your manuscript that highlights changes made to the original version. You should upload this as a separate file labeled 'Revised Manuscript with Track Changes'.
- An unmarked version of your revised paper without tracked changes. You should upload this as a separate file labeled 'Manuscript'.

If you would like to make changes to your financial disclosure, please include your updated statement in your cover letter. Guidelines for resubmitting your figure files are available below the reviewer comments at the end of this letter.

If applicable, we recommend that you deposit your laboratory protocols in protocols.io to enhance the reproducibility of your results. Protocols.io assigns your protocol its own identifier (DOI) so that it can be cited independently in the future. For instructions see: https://journals.plos.org/plosone/s/submission-guidelines#loc-laboratory-protocols. Additionally, PLOS ONE offers an option for publishing peer-reviewed Lab Protocol articles, which describe protocols hosted on protocols.io. Read more information on sharing protocols at https://plos.org/protocols?utm_medium=editorial-email&utm_source=authorletters&utm_campaign=protocols.

As the corresponding author, your ORCID iD is verified in the submission system and will appear in the published article. PLOS supports the use of ORCID, and we encourage all coauthors to register for an ORCID iD and use it as well. Please encourage your coauthors to verify their ORCID iD within the submission system before final acceptance, as unverified ORCID iDs will not appear in the published article. *Only*  the individual author can complete the verification step; PLOS staff *cannot*  verify ORCID iDs on behalf of authors.

We look forward to receiving your revised manuscript.

Kind regards,

Amel Mohamed El Asely

Academic Editor

PLOS One

**Journal Requirements:**

1. If the reviewer comments include a recommendation to cite specific previously published works, please review and evaluate these publications to determine whether they are relevant and should be cited. There is no requirement to cite these works unless the editor has indicated otherwise.

2. Please review your reference list to ensure that it is complete and correct. If you have cited papers that have been retracted, please include the rationale for doing so in the manuscript text, or remove these references and replace them with relevant current references. Any changes to the reference list should be mentioned in the rebuttal letter that accompanies your revised manuscript. If you need to cite a retracted article, indicate the article’s retracted status in the References list and also include a citation and full reference for the retraction notice.

[Note: HTML markup is below. Please do not edit.]

Reviewers' comments:

Reviewer's Responses to Questions

**Comments to the Author**

Reviewer #1: All comments have been addressed

Reviewer #2: (No Response)

**********

2. Is the manuscript technically sound, and do the data support the conclusions?

Reviewer #1: Yes

Reviewer #2: Yes

**********

3. Has the statistical analysis been performed appropriately and rigorously?

Reviewer #1: Yes

Reviewer #2: Yes

**********

4. Have the authors made all data underlying the findings in their manuscript fully available?

The PLOS Data policy

Reviewer #1: Yes

Reviewer #2: Yes

**********

5. Is the manuscript presented in an intelligible fashion and written in standard English?

Reviewer #1: Yes

Reviewer #2: Yes

**********

**Reviewer #1:**  I thank the authors for their responses and for the care taken in revising the manuscript. I believe that the clarifications provided and the revisions implemented have significantly improved the clarity and scientific quality of the work. However, one issue still remains in lines 264–267 concerning the interpretation of the results presented in Figure 3. According to the statistical analysis shown in Figure 3, G. bimaculatus is assigned the letters “a,b”, indicating no significant differences in α-tocopherol content compared with either sesame oil and S. frugiperda (letter “a”) or S. gregaria L5 and S. icipe (letter “b”). Therefore, it is not appropriate to state that G. bimaculatus exhibited higher α-tocopherol levels than sesame oil.

**Reviewer #2:** Attached are my comments about manuscript number: Review PONE-D-26-01997R1

Article entitled “Omega-3 fatty acids and bioactives in aqueous-extracted oils from seven edible insects”

I appreciate the opportunity to review this manuscript. After examining this new version, I note that the authors have taken our suggestions into account, which were intended to improve and refine their manuscript. There are a few areas that could be improved.

1. Throughout the manuscript, the authors have used several abbreviations (w-3 FAs, FAs, FAMEs, ALA, EPA, DHA, SFAs, MUFAs, and PUFAs). However, these are not used consistently throughout the manuscript, and in some cases, the authors repeat the full names. For example, in lines 214, 251, 252–255, 313, 317, 328, and 329.

2 In my opinion, there is no need to repeat the ANOVA followed by the Student-Newman-Keuls (SNK) post hoc test in the “Data Analysis” section. The authors could have included the concentrations of individual fatty acids and flavonoids, as well as oil yields and vitamin E concentrations, in the same paragraph.

3 Although the authors have made some changes to Table 2. Fatty acid composition (μ 221 g/mg of insect oil*) analyzed by GC-MS, further modifications are necessary: 1) the text still needs to be adjusted to fit within the cell; in most cases, the letters used to indicate differences between groups and some numbers are carried over to the next line, making it difficult to correctly read the values and differences described in the table. 2) Some values are formatted as superscripts, while some of the letters used are not formatted as superscripts.

**********

what does this mean?). If published, this will include your full peer review and any attached files.

If you choose “no”, your identity will remain anonymous but your review may still be made public.

**Do you want your identity to be public for this peer review?**  For information about this choice, including consent withdrawal, please see our Privacy Policy

Reviewer #1: No

Reviewer #2: **Yes:** Salvador Atilano Miguel

**********

[NOTE: If reviewer comments were submitted as an attachment file, they will be attached to this email and accessible via the submission site. Please log into your account, locate the manuscript record, and check for the action link "View Attachments". If this link does not appear, there are no attachment files.]

To ensure your figures meet our technical requirements, please review our figure guidelines: https://journals.plos.org/plosone/s/figures

You may also use PLOS’s free figure tool, NAAS, to help you prepare publication quality figures: https://journals.plos.org/plosone/s/figures#loc-tools-for-figure-preparation.

NAAS will assess whether your figures meet our technical requirements by comparing each figure against our figure specifications.

Attachment Submitted filename: Review PONE-D-26-01997R1.docx

# Author comment: Author response to Decision Letter 2

**DOI:** 10.1371/journal.pone.0356807.r004

8 Jun 2026

Omega-3 fatty acids and bioactives in aqueous-extracted oils from seven edible insects

Point-by-Point Response to Reviewers’ Comments

Dear Prof. Amel Mohamed El Asely

The authors are grateful for the valuable input in the manuscript by reviewers and editors. The important changes made include the following: revision of some parts of the introduction, results, discussion and conclusion sections and the correction of grammatical and syntax errors. Below is the list of point-by-point responses to the reviewer’s comments.

Line/Section Comments Authors’ response/action Text revised

Reviewer # 1

Results I thank the authors for their responses and for the care taken in revising the manuscript. I believe that the clarifications provided and the revisions implemented have significantly improved the clarity and scientific quality of the work. However, one issue still remains in lines 264–267 concerning the interpretation of the results presented in Figure 3. According to the statistical analysis shown in Figure 3, G. bimaculatus is assigned the letters “a,b”, indicating no significant differences in α-tocopherol content compared with either sesame oil and S. frugiperda (letter “a”) or S. gregaria L5 and S. icipe (letter “b”). Therefore, it is not appropriate to state that G. bimaculatus exhibited higher α-tocopherol levels than sesame oil We thank the reviewer for this observation. We agree that G. bimaculatus (a,b) was not significantly different from either sesame oil and S. frugiperda L5 (a) or S. gregaria L5 and S. icipe (b), and therefore our original wording could imply an unsupported significant difference. The text has been revised to clarify that only S. gregaria L5 and S. icipe had significantly higher α-tocopherol concentrations than sesame oil, while G. bimaculatus showed an intermediate concentration that was not significantly different from either group. 259-264

Reviewer # 2

Entire manuscript I appreciate the opportunity to review this manuscript. After examining this new version, I note that the authors have taken our suggestions into account, which were intended to improve and refine their manuscript. There are a few areas that could be improved.

1. Throughout the manuscript, the authors have used several abbreviations (w-3 FAs, FAs, FAMEs, ALA, EPA, DHA, SFAs, MUFAs, and PUFAs). However, these are not used consistently throughout the manuscript, and in some cases, the authors repeat the full names. For example, in lines 214, 251, 252–255, 313, 317, 328, and 329.

We thank the reviewer for the positive assessment of our revised manuscript and appreciate the constructive feedback aimed at further improving its clarity and quality.

The mentioned abbreviations have been standardized and corrected throught the manuscript Lines 29, 30,56, 62, 77, 84, 90, 131, 215, 230, 231, 252, 253, 256, 288, 305, 318, 322, 333, 337, 338, 343, 344, 350, 391, 395, 396

Data Analysis 2. In my opinion, there is no need to repeat the ANOVA followed by the Student-Newman-Keuls (SNK) post hoc test in the “Data Analysis” section. The authors could have included the concentrations of individual fatty acids and flavonoids, as well as oil yields and vitamin E concentrations, in the same paragraph We thank the reviewer for the suggestion and have revised the Data Analysis section to remove redundancy and improve clarity, while ensuring all reported variables are clearly aligned with the corresponding results tables and text. Line 192-197

Results 3. Although the authors have made some changes to Table 2. Fatty acid composition (μ 221 g/mg of insect oil*) analyzed by GC-MS, further modifications are necessary: 1) the text still needs to be adjusted to fit within the cell; in most cases, the letters used to indicate differences between groups and some numbers are carried over to the next line, making it difficult to correctly read the values and differences described in the table. 2) Some values are formatted as superscripts, while some of the letters used are not formatted as superscripts Thank you the revisions have been effected in the Table 2 Line 223

Attachment Submitted filename: Response_to_Reviewers_auresp_2.docx

# Peer review: Decision Letter 2

**DOI:** 10.1371/journal.pone.0356807.r005

**Authors:** Amel El Asely

29 Jun 2026

Dear Dr.  Cheseto,

Please submit your revised manuscript by Aug 13 2026 11:59PM. If you will need more time than this to complete your revisions, please reply to this message or contact the journal office at plosone@plos.org. When you're ready to submit your revision, log on to https://www.editorialmanager.com/pone/ and select the 'Submissions Needing Revision' folder to locate your manuscript file.

- A letter that responds to each point raised by the academic editor and reviewer(s). You should upload this letter as a separate file labeled 'Response to Reviewers'.
- A marked-up copy of your manuscript that highlights changes made to the original version. You should upload this as a separate file labeled 'Revised Manuscript with Track Changes'.
- An unmarked version of your revised paper without tracked changes. You should upload this as a separate file labeled 'Manuscript'.

If you would like to make changes to your financial disclosure, please include your updated statement in your cover letter. Guidelines for resubmitting your figure files are available below the reviewer comments at the end of this letter.

If applicable, we recommend that you deposit your laboratory protocols in protocols.io to enhance the reproducibility of your results. Protocols.io assigns your protocol its own identifier (DOI) so that it can be cited independently in the future. For instructions see: https://journals.plos.org/plosone/s/submission-guidelines#loc-laboratory-protocols. Additionally, PLOS ONE offers an option for publishing peer-reviewed Lab Protocol articles, which describe protocols hosted on protocols.io. Read more information on sharing protocols at https://plos.org/protocols?utm_medium=editorial-email&utm_source=authorletters&utm_campaign=protocols.

As the corresponding author, your ORCID iD is verified in the submission system and will appear in the published article. PLOS supports the use of ORCID, and we encourage all coauthors to register for an ORCID iD and use it as well. Please encourage your coauthors to verify their ORCID iD within the submission system before final acceptance, as unverified ORCID iDs will not appear in the published article. *Only*  the individual author can complete the verification step; PLOS staff *cannot*  verify ORCID iDs on behalf of authors.

We look forward to receiving your revised manuscript.

Kind regards,

Amel Mohamed El Asely

Academic Editor

PLOS One

Journal Requirements:

If the reviewer comments include a recommendation to cite specific previously published works, please review and evaluate these publications to determine whether they are relevant and should be cited. There is no requirement to cite these works unless the editor has indicated otherwise.

Please review your reference list to ensure that it is complete and correct. If you have cited papers that have been retracted, please include the rationale for doing so in the manuscript text, or remove these references and replace them with relevant current references. Any changes to the reference list should be mentioned in the rebuttal letter that accompanies your revised manuscript. If you need to cite a retracted article, indicate the article’s retracted status in the References list and also include a citation and full reference for the retraction notice.

[Note: HTML markup is below. Please do not edit.]

Reviewers' comments:

Reviewer's Responses to Questions

**Comments to the Author**

Reviewer #1: All comments have been addressed

Reviewer #2: All comments have been addressed

**********

2. Is the manuscript technically sound, and do the data support the conclusions?

Reviewer #1: Yes

Reviewer #2: Yes

**********

3. Has the statistical analysis been performed appropriately and rigorously?

Reviewer #1: Yes

Reviewer #2: Yes

**********

4. Have the authors made all data underlying the findings in their manuscript fully available?

The PLOS Data policy

Reviewer #1: Yes

Reviewer #2: Yes

**********

5. Is the manuscript presented in an intelligible fashion and written in standard English?

Reviewer #1: Yes

Reviewer #2: Yes

**********

Reviewer #1: (No Response)

Reviewer #2: Dear editors:

Attached are my comments about manuscript number: Review PONE-D-26-01997R2

Article entitled “Omega-3 fatty acids and bioactives in aqueous-extracted oils from seven edible insects”

I appreciate the opportunity to review this revised manuscript. After examining this new version, I note that the authors have successfully addressed most of our previous suggestions, which has significantly improved and refined the manuscript. However, there are still some lingering issues regarding Table 2 that require attention before publication.

1.

The formatting remains problematic for specific rows. In particular, the fatty acids corresponding to retention times 19.69, 22.23, 24.06, and 26.28 still exhibit overlapping or combined letters.

2.

To improve the presentation and readability of the data, the authors should consider rounding the decimal values for specific rows. This applies particularly to the fatty acid values at retention times 30.06, 24.94, and 25.40, as well as the rows for Total PUFAs and n-3 PUFAs. Consolidating these numbers via rounding would prevent text from overflowing to the next line within the cells.

**********

what does this mean?). If published, this will include your full peer review and any attached files.

If you choose “no”, your identity will remain anonymous but your review may still be made public.

**Do you want your identity to be public for this peer review?**  For information about this choice, including consent withdrawal, please see our Privacy Policy

Reviewer #1: No

Reviewer #2: **Yes:** Salvador Atilano Miguel

**********

[NOTE: If reviewer comments were submitted as an attachment file, they will be attached to this email and accessible via the submission site. Please log into your account, locate the manuscript record, and check for the action link "View Attachments". If this link does not appear, there are no attachment files.]

To ensure your figures meet our technical requirements, please review our figure guidelines: https://journals.plos.org/plosone/s/figures

You may also use PLOS’s free figure tool, NAAS, to help you prepare publication quality figures: https://journals.plos.org/plosone/s/figures#loc-tools-for-figure-preparation.

NAAS will assess whether your figures meet our technical requirements by comparing each figure against our figure specifications.

Attachment Submitted filename: Review PONE-D-26-01997R2.pdf

# Author comment: Author response to Decision Letter 3

**DOI:** 10.1371/journal.pone.0356807.r006

3 Jul 2026

Omega-3 fatty acids and bioactives in aqueous-extracted oils from seven edible insects

Point-by-Point Response to Reviewers’ Comments

Dear Prof. Amel Mohamed El Asely

The authors are grateful for the valuable input in the manuscript by reviewers and editors. The important changes made include the following: revision of some parts of the introduction, results, discussion and conclusion sections and the correction of grammatical and syntax errors. Below is the list of point-by-point responses to the reviewer’s comments.

Line/Section Comments Authors’ response/action Text revised

Reviewer # 1

Results I thank the authors for their responses and for the care taken in revising the manuscript. I believe that the clarifications provided and the revisions implemented have significantly improved the clarity and scientific quality of the work. However, one issue still remains in lines 264–267 concerning the interpretation of the results presented in Figure 3. According to the statistical analysis shown in Figure 3, G. bimaculatus is assigned the letters “a,b”, indicating no significant differences in α-tocopherol content compared with either sesame oil and S. frugiperda (letter “a”) or S. gregaria L5 and S. icipe (letter “b”). Therefore, it is not appropriate to state that G. bimaculatus exhibited higher α-tocopherol levels than sesame oil We thank the reviewer for this observation. We agree that G. bimaculatus (a,b) was not significantly different from either sesame oil and S. frugiperda L5 (a) or S. gregaria L5 and S. icipe (b), and therefore our original wording could imply an unsupported significant difference. The text has been revised to clarify that only S. gregaria L5 and S. icipe had significantly higher α-tocopherol concentrations than sesame oil, while G. bimaculatus showed an intermediate concentration that was not significantly different from either group. 259-264

Reviewer # 2

Entire manuscript I appreciate the opportunity to review this manuscript. After examining this new version, I note that the authors have taken our suggestions into account, which were intended to improve and refine their manuscript. There are a few areas that could be improved.

1. Throughout the manuscript, the authors have used several abbreviations (w-3 FAs, FAs, FAMEs, ALA, EPA, DHA, SFAs, MUFAs, and PUFAs). However, these are not used consistently throughout the manuscript, and in some cases, the authors repeat the full names. For example, in lines 214, 251, 252–255, 313, 317, 328, and 329.

We thank the reviewer for the positive assessment of our revised manuscript and appreciate the constructive feedback aimed at further improving its clarity and quality.

The mentioned abbreviations have been standardized and corrected throught the manuscript Lines 29, 30,56, 62, 77, 84, 90, 131, 215, 230, 231, 252, 253, 256, 288, 305, 318, 322, 333, 337, 338, 343, 344, 350, 391, 395, 396

Data Analysis 2. In my opinion, there is no need to repeat the ANOVA followed by the Student-Newman-Keuls (SNK) post hoc test in the “Data Analysis” section. The authors could have included the concentrations of individual fatty acids and flavonoids, as well as oil yields and vitamin E concentrations, in the same paragraph We thank the reviewer for the suggestion and have revised the Data Analysis section to remove redundancy and improve clarity, while ensuring all reported variables are clearly aligned with the corresponding results tables and text. Line 192-197

Results 3. Although the authors have made some changes to Table 2. Fatty acid composition (μ 221 g/mg of insect oil*) analyzed by GC-MS, further modifications are necessary: 1) the text still needs to be adjusted to fit within the cell; in most cases, the letters used to indicate differences between groups and some numbers are carried over to the next line, making it difficult to correctly read the values and differences described in the table. 2) Some values are formatted as superscripts, while some of the letters used are not formatted as superscripts Thank you the revisions have been effected in the Table 2 Line 223

Attachment Submitted filename: Response_to_Reviewers_auresp_3.docx

# Peer review: Decision Letter 3

**DOI:** 10.1371/journal.pone.0356807.r007

**Authors:** Amel El Asely

10 Aug 2026

Omega-3 fatty acids and bioactives in aqueous-extracted oils from seven edible insects

PONE-D-26-01997R3

Dear Dr. Cheseto,

We’re pleased to inform you that your manuscript has been judged scientifically suitable for publication and will be formally accepted for publication once it meets all outstanding technical requirements.

Within one week, you’ll receive an e-mail detailing the required amendments. When these have been addressed, you’ll receive a formal acceptance letter and your manuscript will be scheduled for publication.

An invoice will be generated when your article is formally accepted. Please note, if your institution has a publishing partnership with PLOS and your article meets the relevant criteria, all or part of your publication costs will be covered. Please make sure your user information is up-to-date by logging into Editorial Manager at Editorial Manager® and clicking the ‘Update My Information' link at the top of the page. For questions related to billing, please contact billing support.

If your institution or institutions have a press office, please notify them about your upcoming paper to help maximize its impact. If they’ll be preparing press materials, please inform our press team as soon as possible -- no later than 48 hours after receiving the formal acceptance. Your manuscript will remain under strict press embargo until 2 pm Eastern Time on the date of publication. For more information, please contact onepress@plos.org.

Kind regards,

Amel Mohamed El Asely

Academic Editor

PLOS One

Additional Editor Comments (optional):

Reviewers' comments:

Reviewer's Responses to Questions

**Comments to the Author**

Reviewer #1: All comments have been addressed

Reviewer #2: All comments have been addressed

**********

2. Is the manuscript technically sound, and do the data support the conclusions?

Reviewer #1: Yes

Reviewer #2: Yes

**********

3. Has the statistical analysis been performed appropriately and rigorously?

Reviewer #1: Yes

Reviewer #2: Yes

**********

4. Have the authors made all data underlying the findings in their manuscript fully available?

The PLOS Data policy

Reviewer #1: Yes

Reviewer #2: Yes

**********

5. Is the manuscript presented in an intelligible fashion and written in standard English?

Reviewer #1: Yes

Reviewer #2: Yes

**********

Reviewer #1: (No Response)

Reviewer #2: Dear Editors:

Please find attached my comments on manuscript no.: Review PONE-D-26-01997R3

Article titled “Omega-3 fatty acids and bioactives in aqueous-extracted oils from seven edible

insects”

I appreciate the opportunity to review this revised manuscript. After reviewing this new version, I note that the authors have made the suggested changes. Therefore, I approve the current version and recommend the manuscript for publication.

**********

what does this mean?). If published, this will include your full peer review and any attached files.

If you choose “no”, your identity will remain anonymous but your review may still be made public.

**Do you want your identity to be public for this peer review?**  For information about this choice, including consent withdrawal, please see our Privacy Policy

Reviewer #1: No

Reviewer #2: **Yes:** Salvador Atilano Miguel

**********

Attachment Submitted filename: Review PONE-D-26-01997R3.pdf

# Editor report: Acceptance letter

**DOI:** 10.1371/journal.pone.0356807.r008

**Authors:** Amel El Asely

PONE-D-26-01997R3

PLOS One

Dear Dr. Cheseto,

I'm pleased to inform you that your manuscript has been deemed suitable for publication in PLOS One. Congratulations! Your manuscript is now being handed over to our production team.

At this stage, our production department will prepare your paper for publication. This includes ensuring the following:

* All references, tables, and figures are properly cited

* All relevant supporting information is included in the manuscript submission,

* There are no issues that prevent the paper from being properly typeset

You will receive further instructions from the production team, including instructions on how to review your proof when it is ready. Please keep in mind that we are working through a large volume of accepted articles, so please give us a few days to review your paper and let you know the next and final steps.

Lastly, if your institution or institutions have a press office, please let them know about your upcoming paper now to help maximize its impact. If they'll be preparing press materials, please inform our press team within the next 48 hours. Your manuscript will remain under strict press embargo until 2 pm Eastern Time on the date of publication. For more information, please contact onepress@plos.org.

You will receive an invoice from PLOS for your publication fee after your manuscript has reached the completed accept phase. If you receive an email requesting payment before acceptance or for any other service, this may be a phishing scheme. Learn how to identify phishing emails and protect your accounts at https://explore.plos.org/phishing.

If we can help with anything else, please email us at customercare@plos.org.

Thank you for submitting your work to PLOS One and supporting open access.

Kind regards,

PLOS ONE Editorial Office Staff

on behalf of

Prof. Amel Mohamed El Asely

Academic Editor

PLOS One
